# Visualizing Cholesterol in the Brain by On-Tissue Derivatization and Quantitative Mass Spectrometry Imaging

**DOI:** 10.1021/acs.analchem.0c05399

**Published:** 2021-03-09

**Authors:** Roberto Angelini, Eylan Yutuc, Mark F. Wyatt, Jillian Newton, Fowzi A. Yusuf, Lauren Griffiths, Benjamin J. Cooze, Dana El Assad, Gilles Frache, Wei Rao, Luke B. Allen, Zeljka Korade, Thu T. A. Nguyen, Rathnayake A. C. Rathnayake, Stephanie M. Cologna, Owain W. Howell, Malcolm R. Clench, Yuqin Wang, William J. Griffiths

**Affiliations:** † Medical School, Swansea University, Singleton Park, Swansea SA2 8PP, Wales, U.K.; ‡ Centre for Mass Spectrometry Imaging, Biomolecular Research Centre, Sheffield Hallam University, Howard Street, Sheffield S1 1WB, U.K.; § Materials Research and Technology, Luxembourg Institute of Science and Technology, Belvaux L-4422, Luxembourg; ∥ European Application Laboratory, Waters Corporation, Stamford Avenue, Altrincham Road, Wilmslow SK9 4AX, U.K.; ⊥ Departments of Pediatrics and Biochemistry and Molecular Biology, University of Nebraska Medical Center, Omaha, Nebraska 68198, United States; # Department of Chemistry and Laboratory of Integrated Neuroscience, University of Illinois at Chicago, Chicago, Illinois 60607, United States

## Abstract

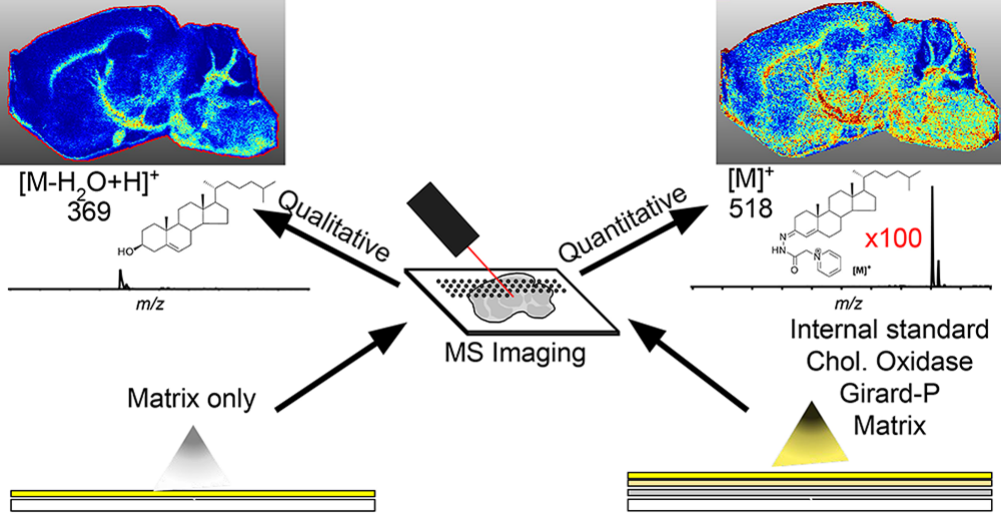

Despite being a critical molecule in the brain, mass spectrometry imaging (MSI) of cholesterol has been under-reported compared to other lipids due to the difficulty in ionizing the sterol molecule. In the present work, we have employed an on-tissue enzyme-assisted derivatization strategy to improve detection of cholesterol in brain tissue sections. We report distribution and levels of cholesterol across specific structures of the mouse brain, in a model of Niemann-Pick type C1 disease, and during brain development. MSI revealed that in the adult mouse, cholesterol is the highest in the pons and medulla and how its distribution changes during development. Cholesterol was significantly reduced in the corpus callosum and other brain regions in the *Npc1* null mouse, confirming hypomyelination at the molecular level. Our study demonstrates the potential of MSI to the study of sterols in neuroscience.

Cholesterol is the most abundant individual molecular species in plasma membranes of animals, accounting for approximately 20–25% of the lipid molecules in the plasma membrane of most cells,^1^ with only a small proportion of cellular cholesterol embedded in organelles. Within membranes, cholesterol influences bilayer fluidity and permeability and lipid and protein sorting in membrane trafficking.^2^ In the brain, cholesterol makes up about 15% of the dry weight of white matter (WM) and is a major component of myelin sheaths.^3^ However, little is known about how sterol concentrations vary in different anatomical locations or at sites of focal pathology.^4^ Cholesterol is metabolized to oxysterols, steroid hormones, and bile acids. These metabolic pathways are at least partially operative in the brain, and their metabolic products and intermediates serve as biologically active signaling molecules.^5^ In light of this, it is not surprising that impairment in sterol homeostasis and signaling is implicated in a number of human disorders including neurodegenerative and neurodevelopmental conditions.^6,7^ Dysregulation of cholesterol homeostasis has been implicated in Alzheimer’s disease^8^ and multiple sclerosis,^9^ while inborn errors of cholesterol biosynthesis, metabolism, and transport can result in neurological disorders,^10^ such as Smith–Lemli–Opitz syndrome (SLOS, 7-dehydrocholesterol reductase deficiency) and Niemann-Pick disease types C1 and C2 (NPC1 and NPC2, respectively).

Traditionally, cholesterol analysis in tissue begins with homogenization followed by lipid extraction, leading to loss of spatial information. To better understand sterol biochemical and physiological roles, there is a need to match molecular abundance with the exact location. To this end, brain dissection can be coupled to gas chromatography (GC)—mass spectrometry (MS) or to liquid chromatography (LC)—MS.^8,11,12^ An alternative method to map sterol concentrations in the brain is by exploiting mass spectrometry imaging (MSI), for example, time-of-flight (ToF) secondary-ion MS (SIMS)—MSI, where cholesterol has been detected with high intensities, even at subcellular resolutions. However, a drawback with this approach is that ToF-SIMS is a surface-sensitive technique, and cholesterol has been shown to migrate to and crystallize on the surface, covering up all colocalizing species in the tissue. Matrix-assisted laser desorption/ionization (MALDI)-MSI has been employed to detect and identify multiple molecular species and simultaneously map their distribution in tissue sections.^13,14^ It can generate pixelated MS data at near-cellular resolution, providing spatial mapping of protein, peptide, and lipid molecules according to X–Y position on a tissue section.^15,16^ MALDI-MSI has been used to image lipids in the brain;^17^ however, cholesterol and other sterols tend to be poorly ionized by conventional MALDI and are discriminated against when compared to lipid classes that are easily ionized. Cholesterol has been detected in MALDI-MSI studies,^18^ but to enhance ionization, other desorption methods have been employed, including nanostructure-initiator MS,^19^ sputtered silver MALDI,^20^ and silver nanoparticle MALDI.^21^ Silver ions coordinate with carbon–carbon double bonds, providing cationic adducts of sterols in the MALDI matrix. Recently, “MALDI-2”-MSI has been developed, where a postdesorption second-tuneable laser enhances the ionization of neutral lipid species including cholesterol, allowing improved visualization in tissue sections.^22^ Alternatively, derivatization strategies can be utilized to enhance sterol ionization. For in-solution studies, we and others have exploited enzyme-assisted derivatization for sterol analysis (EADSA)^23,24^ where the sterol molecule is reacted first with the cholesterol oxidase enzyme to oxidize the 3β-hydroxy group to 3-oxo and then with Girard-P (GP) hydrazine to give a charge-tagged sterol hydrazone (Figure 1). This strategy enhances the MS signal and provides unique structural information upon multistage fragmentation (MS^*n*^) which, together with the retention time and accurate mass measurements, can provide unambiguous identification, even of isomeric species. Of note, others have similarly exploited a Girard-T hydrazine to derivatize and visualize by MSI steroid molecules, already possessing an oxo function.^25,26^

**Figure 1:**
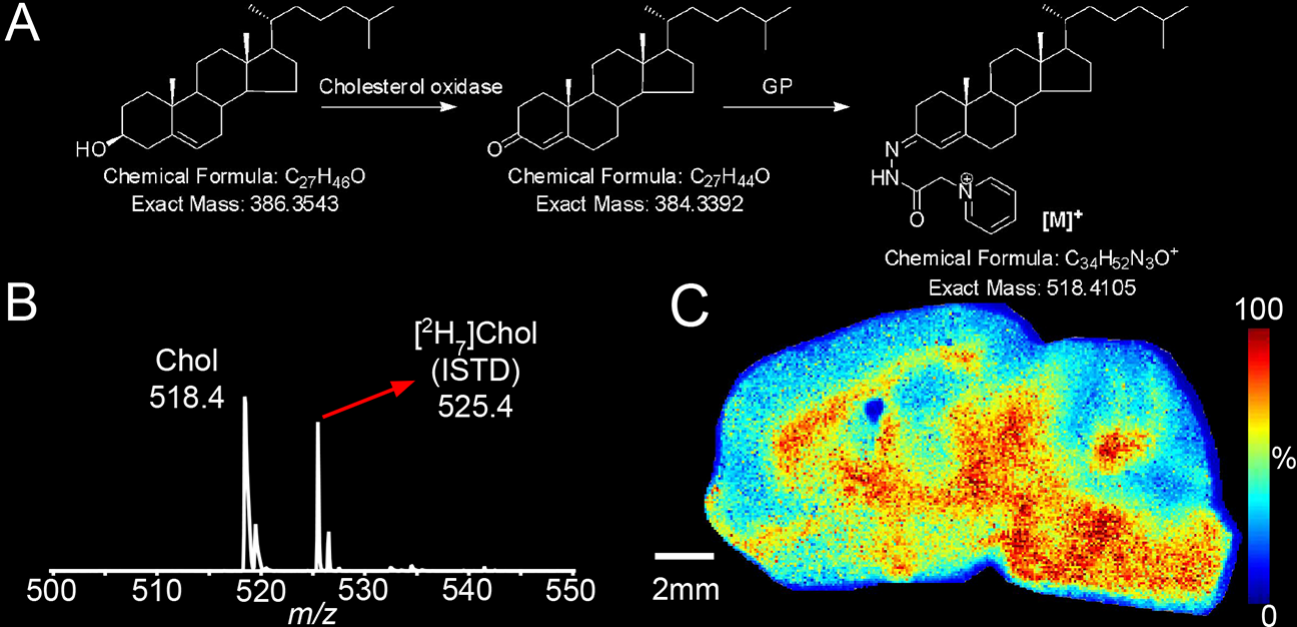
MSI of cholesterol in WT mouse brain exploiting on-tissue EADSA. (A) EADSA process occurs in two steps where the 3β-hydroxy-5-ene group is first converted to a 3-oxo-4-ene by the enzyme cholesterol oxidase and then charge-tagged with the GP hydrazine. (B) Typical mass spectrum generated in an EADSA-MALDI-MSI experiment for a single pixel. The spectrum, in the *m*/*z* range 500–550, is dominated by the signals of derivatized endogenous cholesterol at *m*/*z* 518.4 and sprayed-on standard [^2^H_7_]cholesterol at *m*/*z* 525.4. *In each pixel*, the peak at 518.4 is normalized to the peak at 525.4, and an MS image of the distribution of cholesterol across the mouse brain tissue section is created as shown in (C). MSI data were acquired on a vacuum-MALDI-TOF MS at a pixel size of 50 μm and visualized with an isolation window width of 0.5 *m*/*z*. See Supporting Information, Table S1 for instruments used in each figure.

We have adapted EADSA to MSI in order to image cholesterol in the developing and adult mouse brain and in a mouse model of Niemann-Pick C1 disease (*Npc1 null*) at 30–50 μm pixel size. We demonstrate the use of isotope-labeled standards to determine the absolute quantity of cholesterol in different anatomical regions of the mouse brain. A quantitative MSI of the adult wild-type (WT) mouse in sagittal sections was determined by identifying the pons and medulla of the brain stem as the regions with the highest cholesterol level. The WT mouse was compared to the *Npc1 null* mouse showing a significant reduction of cholesterol in the corpus callosum. In the WT mouse brain at birth, cholesterol is the highest in the pontine hind brain that will develop into the cholesterol-rich pons region in the adult mouse. The derivatization-based method has potential to be expanded to low abundance sterols, while simultaneously detecting other nonderivatized lipid classes.

## Materials and Methods

The aim of the study was to develop an MSI method suitable to map the distribution and to determine the concentration of cholesterol in different anatomical regions of mouse brain.

### Chemicals and Reagents

HPLC-grade methanol, propan-2-ol, acetonitrile, ethanol, xylene, and industrial methylated spirit were from Fisher Scientific (Loughborough, UK). Glacial acetic acid was from VWR (Lutterworth, UK). [25,26,26,26,27,27,27-^2^H_7_]Cholesterol was from Avanti Polar Lipids (Alabaster, AL). Cholesterol oxidase from *Streptomyces* sp., and potassium dihydrogen phosphate, Luxol Fast Blue (LFB), Cresyl Violet (CV), DPX mountant, paraformaldehyde (PFA), lithium carbonate, and α-cyano-4-hydroxycinnamic acid (CHCA) were from Merck (Dorset, UK). GP-hydrazine was from TCI (Zwijndrecht, Belgium).

### Experimental Models

In the present study, adult WT and *Npc1*^–/–^ mice and the phenotypically normal newborn *Dhcr7*^T93M/+^ mouse were employed. Details can be found in Supporting Information Methods.

### Tissue Sectioning

Brain tissue, mounted on and only partially embedded in the optimal cutting temperature (OCT) compound, was cryosectioned using a Leica Cryostat CM1900 (Leica Microsystems, Milton Keynes, UK) at a chamber temperature of −18 °C into 10 μm-thick sections which were thaw-mounted onto optical microscope slides for histology or onto indium tin oxide (ITO)-coated glass slides for MSI and stored at −80 °C until use. ITO-coated glass slides (8–12 Ohm/Sq) were from Diamond Coatings (Halesowen, UK). Three sections were mounted on each glass slide, and each section was separated by 100 μm from the adjacent section, that is, the nine sections in between were placed on other consecutive slides.

### Histology

Tissue sections adjacent to sections analyzed by MSI were thawed, fixed in PFA to preserve anatomy, and subjected to LFB histology with cresyl violet as the counterstain^27^ (Supporting Information Methods). Histological data were analyzed by QuPath^28^ and ImageJ (NIH) following whole-section digitization at 400× magnification using a Zeiss AxioScanner.

### Region of Interest (ROI) Analysis and Quantitative Morphometry

Quantitative analysis of histological data was carried out as follows. To assess fiber myelination, the caudate-putamen ROI was outlined on the digitized images with QuPath. Images of defined ROI were cropped and converted into an 8-bit format with ImageJ to mark and measure specific areas. The threshold was adjusted to exclude cell nuclei and automatically outline WM areas exclusively, and the total WM area was measured per section. These data were used to calculate the percentage of myelinated fibers in the selected ROI. Cerebellar area and length of the Purkinje cell layer were also defined with QuPath, and Purkinje cells were manually counted to determine cell linear density.

### Stereology

Stereological methods were employed to identify ROI within mouse brain sagittal tissue sections. The defined ROI was employed to analyze both the MSI and the histology data. For determination of ROI, we referred to the Allen Mouse Brain Atlas (AMBA, Allen Institute for Brain Science) (sagittal sections, P56, https://atlas.brain-map.org/atlas?atlas=2).^29^ Details can be found in Supporting Information Methods.

### Deposition of the Standard and On-Tissue EADSA

This was performed as described in ref (30) with minor modifications. Mouse brain sections mounted on an ITO-coated glass slide were transferred from a −80 °C freezer to a vacuum desiccator. After 15 min dessication, [^2^H_7_]cholesterol (200 ng/μL in ethanol) was sprayed from a SunCollect sprayer (SunChrom, Friedrichsdorf, Germany, supplied by KR Analytical Ltd, Cheshire, UK) at a flow rate of 20 μL/min at a linear velocity of 900 mm/min with a 2 mm line distance and height of 30 mm from the section in a series of 18 layers. The resulting density of the deuterated standard was 40 ng/mm^2^ (see below). The sprayer was thoroughly flushed with about 2 mL of methanol after which cholesterol oxidase (0.264 units/mL in 100 μM KH_2_PO_4_ pH 7) was sprayed for 18 layers. The first layer was applied at 10 μL/min, the second was applied at 15 μL/min, and then all the subsequent layers were applied at 20 μL/min to give an enzyme density of 0.05 munits/mm^2^. Thereafter, the enzyme-coated slide was placed on a PTFE bed in a glass jar (11 cm × 11 cm × 7.5 cm) above 30 mL of warm water (37 °C) and then incubated at 37 °C for 1 h. The slide was removed, and the tissue was dried in a vacuum desiccator for 15 min. GP (5 mg/mL in 70% methanol, 5% acetic acid) was sprayed on the dried slide with the same spray parameters as used for spraying of cholesterol oxidase. The resulting GP density was 1.00 μg/mm^2^. The slide was then placed in the custom-made humidity chamber as mentioned above containing 10 mL of prewarmed (37 °C) 50% methanol and 5% acetic acid and incubated at 37 °C for 1 h. The slide was removed and dried in a vacuum desiccator and then stored in a cold room (4 °C) until MSI analyses. Desorption electrospray ionization (DESI)-MSI experiments were performed without any further pretreatment. For MALDI-MSI, on the next day, the desiccator was allowed to reach room temperature, and then, the slide was removed and sprayed with the CHCA matrix. CHCA was sprayed from an HTX TM-sprayer (HTX Technologies, NC, USA) at 5 mg/mL in water/propan-2-ol/acetonitrile (3:4:3, v/v/v) at a flow rate of 80 μL/min and a linear velocity of 1200 mm/min, with 2 mm line distance and a criss-cross deposition method which alternates vertical and horizontal passes, for a total of 8, with an offset of 1 mm, resulting in a matrix density of 1.33 μg/mm^2^. The sprayer nozzle was heated at 70 °C to enhance the solvent evaporation rate.

### Mass Spectrometry Imaging

Following EADSA treatment, tissues sections were analyzed using different mass spectrometers. Optimized instrumental parameters are described below.

### Vacuum MALDI-TOF-MSI

Experiments were carried out on an ultrafleXtreme MALDI TOF/TOF mass spectrometer (Bruker Daltonics, Bremen, Germany) equipped with a Smartbeam Nd:YAG laser emitting at 355 nm (2 kHz) and operated in the reflectron mode and positive polarity. Each mass spectrum was automatically acquired using the autoexecute method in FlexControl (Bruker) software in the *m*/*z* range of 400–1000. The pixel size was set at 50 μm using flexImaging 4.1 software (Bruker), setting laser focus to “small”. The laser spot size was about 50 μm, according to factory specifications and as verified by visual inspection with the instrument camera. Each raster was sampled with 200 shots in five steps for a total of 1000 shots per raster. The total acquisition time was typically about 11.5 h for a total of ∼27,000 positions. The MALDI instrument was calibrated using a mixture of phosphatidylcholine and lysophosphatidylcholine (Avanti Polar Lipids). After measurement, imaging spectra were recalibrated using the batch process in flexAnalysis. On-tissue, mass accuracy was typically within ∼100 ppm of the theoretical mass. Data were analyzed and visualized using flexImaging 3.0 (Bruker) and SCiLS Lab 2014b (SCiLS, Bremen, Germany) without any processing step. Data were visualized using normalization to [^2^H_7_]cholesterol at *m*/*z* 525.5. Mass selection windows for ions of interests were chosen with a width of ±0.25 Da in flexImaging 3.0 and of ±0.125% in SCiLS Lab 2014b. A mass resolution of 20,000 (fwhm) was typically achieved in a single pixel. An optical image of each tissue section was acquired prior to the MS acquisition by means of a flatbed scanner.

### AP-MALDI-MSI

MSI was carried out in the positive-ion mode with an Orbitrap Elite hybrid linear ion trap (LIT)-Orbitrap mass spectrometer (ThermoFisher Scientific) coupled with an AP-MALDI UHR source (MassTech, Maryland USA, supplied by KR Analytical Ltd) equipped with a Nd:YAG laser emitting at 355 nm. Full scan (MS^1^) imaging analysis was performed with *m*/*z* measurement in the Orbitrap over the *m*/*z* range of 400–1200 at 60,000 resolution (fwhm at *m*/*z* 400), MALDI laser energy was set at 45% of the maximum, and frequency was 1.5 kHz. Data were acquired in the constant speed raster (CSR) mode at a scan speed of 2.8 mm/min and a pixel size of 30 μm. A lock mass for [^2^H_7_]cholesterol at *m*/*z* 525.4544 was employed. The acquisition of one mouse brain tissue section was achieved in about 15 h. In MS^3^ experiments, the MALDI laser energy was set at 14% and frequency was 1.5 kHz. Data were acquired in the CSR mode at a scan speed of 3 mm/min and a pixel size of 40 μm. In the LIT, precursor ions were isolated and fragmented with an isolation width of 2 and an arbitrary collision-induced dissociation (CID) energy of 35%. The most intense fragment ion produced in MS^2^ was selected with an isolation width of 2 and fragmented with a CID energy of 40% to produce an MS^3^ spectrum. MS^3^ spectra of cholesterol and [^2^H_7_]cholesterol were acquired in each pixel. The MS^3^ transitions for cholesterol and [^2^H_7_]cholesterol were 518.4 → 439.4→ and 525.4 → 446.4 →, respectively.

Data were analyzed and visualized using ImageQuest (ThermoFisher Scientific). Alternatively, after exporting the file into an imzml format, data were analyzed by SCiLS Lab MVS 2014c (SCiLS, Bremen, Germany) without any processing step. MS^1^ data were normalized to [^2^H_7_]cholesterol at *m*/*z* 525.454. Ions of interests were extracted with a width of 7 mDa and 0.3 Da for MS^1^ and MS^3^ scans, respectively.

### Vacuum-MALDI-Q-IM-TOF MSI

Experiments were carried out on two Synapt G2-Si instruments (Waters, Wilmslow, UK) exploiting Waters HDI 1.4 software for acquisition and image visualization. Images were generated from spectra acquired in the positive-ion mode in the *m*/*z* range 400–1000. The laser frequency was 1 kHz, and power was kept at 100 arbitrary units. The scan time was 0.5 s, and the pixel size was 50 μm. IMS cell wave velocity was from 1000 to 300 m/s, and transfer wave velocity was 281 m/s. In all experiments, the cholesterol signal was measured to better than 5 ppm mass accuracy.

### DESI-Q-IM-TOF MSI

Experiments were carried out on a Synapt G2-Si. Spectra were acquired in the positive ion mode in the *m*/*z* range 100–1200, with a needle voltage of 4.5 kV. The DESI solvent flow rate was 1.25 μL/min. The scan time was 0.25 s, and the pixel size was 25 μm. IMS cell wave velocity was from 1000 to 300 m/s, and transfer wave velocity was 281 m/s. In all experiments, the cholesterol signal was measured with a mass accuracy better than 8 ppm.

### Quantification

Known amounts of [^2^H_7_]cholesterol were sprayed on tissue prior to the EADSA process. This procedure corrects for variation in the matrix effect and MS response. The linearity of the on-tissue response of sprayed-on [^2^H_7_]cholesterol verses endogenous cholesterol was determined by spraying eight consecutive tissue sections with [^2^H_7_]cholesterol at varying densities (endogenous cholesterol areal density is assumed to be constant for a given ROI across the consecutive slices). Quantification was made from [M]^+^ ion signal intensities, averaged in each brain region. Brain regions of interest were defined according to AMBA. The areal density of cholesterol in defined regions of interest was calculated by correlating signal intensity to that of known density of [^2^H_7_]cholesterol sprayed on tissue. All quantitative measurements were analyzed employing SCiLS Lab MVS 2019c (SCilS, Bremen, Germany).

### Statistics

To determine statistical difference in cholesterol areal density between defined regions of interest in five adult WT mice, two-way ANOVA was performed with cholesterol areal density as the dependent variable and the mouse and brain region as factors. The interaction between the mouse and brain region was used as error variance. The residuals representing the interaction deviations were approximately normally distributed. Tukey’s multiple comparisons test was used to identify significant differences between brain regions. Statistical analysis was applied for the assessment of myelinated fiber density and specific cell counts, in defined brain regions of WT and *Npc1*^*–/–*^ mouse brain. Five WT and three *Npc1*^*–/–*^ brains were employed, analyzing three or more sections for each mouse.

To determine statistical differences in cholesterol areal density in defined regions of interest between WT and *Npc1*^–/–^ mouse brain, a Shapiro–Wilk test for normality was performed, followed by an unpaired *t*-test for significance. The analyses were performed using GraphPad Prism 8.2.1 software (GraphPad Software, Inc, CA, USA). A *P*-value of less than 0.05 was considered statistically significant. *P* < 0.05, *; *P* < 0.01, **; and *P* < 0.001, ***. All whiskers on bar graphs represent 1 standard deviation. Note that one of the five control mice was not considered in the calculation of the average cholesterol areal density for the caudate-putamen as it was not sectioned on an equivalent anatomical plane.

## Results and Discussion

In MALDI-MS and electrospray ionization (ESI)-MS, cholesterol is poorly ionized and is often detected as the ammonium adduct [M + NH_4_]^+^ at *m*/*z* 404.39 or as the dehydrated protonated molecule [M + H–H_2_O]^+^ at *m*/*z* 369.35.^31^ In this study, to enhance ionization of sterols, we exploit the EADSA method, previously used for in-solution analysis of sterols. Once the sterol analyte is specifically and effectively charge-tagged by EADSA (Figure 1A), it is readily analyzed by MSI, thereby allowing its detection and identification (e.g., by MS^3^) and the mapping of its distribution. The advantage of this methodology is fourfold in that it (i) greatly increases sensitivity, (ii) allows for absolute quantification, (iii) enhances structural information, and, equally importantly, (iv) increases analytical specificity. Here, we report how EADSA has been adapted to work on brain tissue sections for MSI studies.

### Quantitative MSI of Sterols in WT Mouse Brain

Initial studies were performed using sagittal mouse brain sections with a MALDI-TOF instrument. GP-tagged cholesterol gives an intense [M]^+^ signal, as does sprayed-on [^2^H_7_]cholesterol, and dominates the resulting mass spectrum (Figure 1B). An MS image of cholesterol distribution, normalized *in each pixel* to [^2^H_7_]cholesterol sprayed-on standard, is shown in Figure 1C.

**Figure 2:**
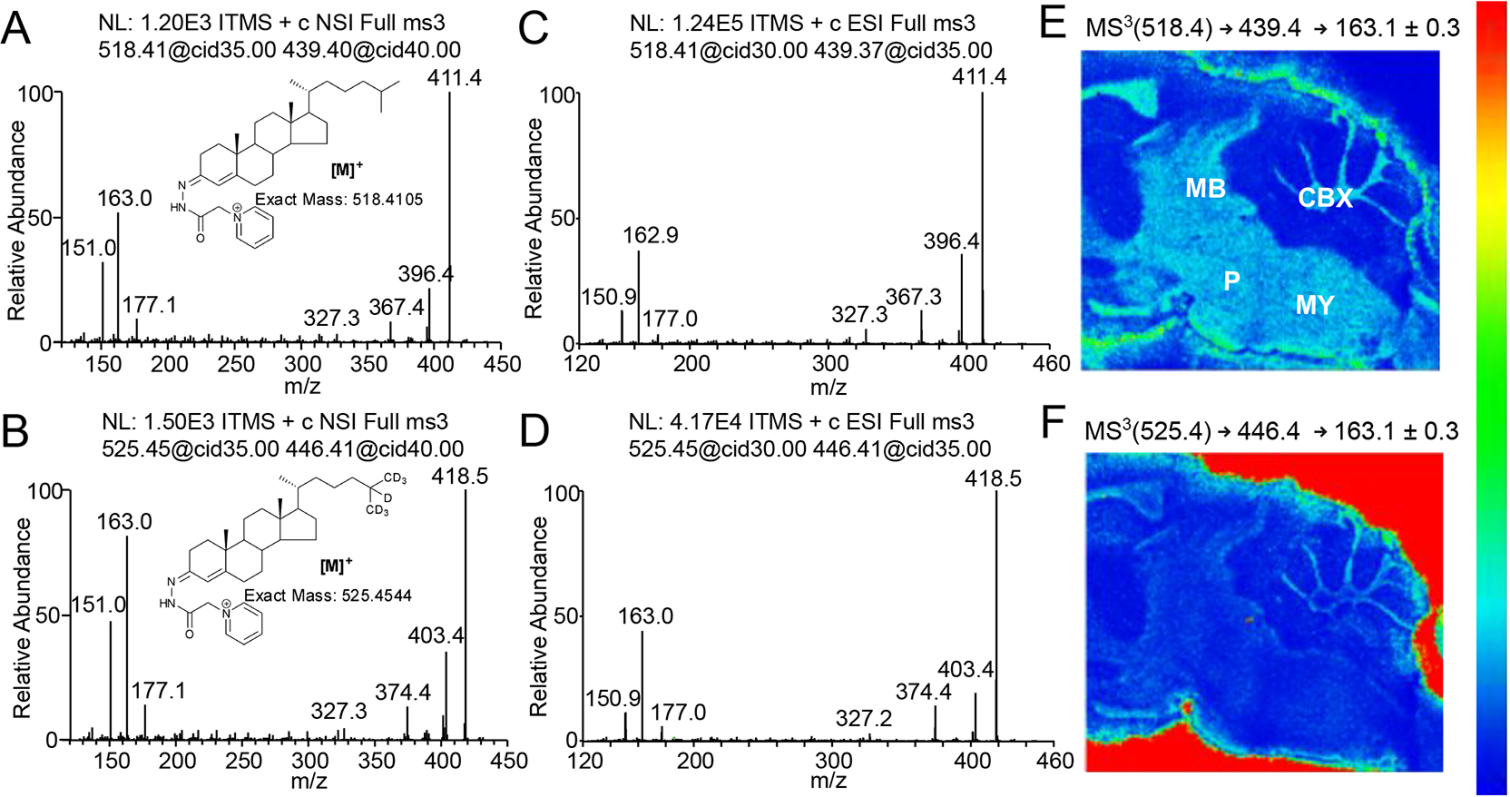
MS^3^ fragmentation patterns of (A) tissue-endogenous cholesterol and (B) sprayed-on [^2^H_7_]cholesterol, in a single pixel obtained in the LIT of an AP-MALDI-Orbitrap Elite after on-tissue EADSA of a mouse brain tissue section and of (C) cholesterol and (D) [^2^H_7_]cholesterol reference standards obtained in the LIT of an ESI-Orbitrap Elite following in-solution EADSA. The MS^3^ spectra were obtained for the transitions [M]^+^ → [M – Py]^+^→. (E,F) MSI of the distribution of the MS^3^ fragment ion at *m*/*z* 163 from (E) cholesterol and (F) [^2^H_7_]cholesterol in a sagittal brain section of WT adult mouse.

To confirm the identity of the signals assigned to cholesterol, we separately carried out MS^3^ ([M]^+^ → [M – Py]^+^→, where Py corresponds to the pyridine component of the GP-tag, see Supporting Information, Figure S1) analysis of the peaks at *m*/*z* 518.41 (cholesterol) and *m*/*z* 525.45 ([^2^H_7_]cholesterol) using AP-MALDI on an Orbitrap Elite, see Figure 2. In Supporting Information, Figure S1, structures of the major fragment ions observed in Figure 2 are described. The fragment ion at *m*/*z* 163 (*b_3_-28) is formed by cleavage of the A/B ring and is devoid of the CD rings and the side chain. It is present in MS^3^ spectra of both cholesterol (Figure 2C) and [^2^H_7_]cholesterol (Figure 2D) authentic standards and can thus be exploited in a multiple reaction monitoring (MRM)-like experiment to confirm the location of tissue-endogenous cholesterol and sprayed-on [^2^H_7_]cholesterol in each pixel (Figure 2A,B, respectively). Figure 2E shows that the MRM transition 518.4 → 439.4 → 163 is essentially absent off tissue, while most notably enriched in the midbrain, pons, medulla, and WM tracts of the cerebellum. Conversely, Figure 2F shows that the MRM transition 525.5 → 446.4 → 163 is saturated off tissue, while being quite evenly distributed on tissue. This transition does show some variation on tissue due to matrix effects. For MS^3^ data, the current software does not allow automated normalization of cholesterol signals to the internal standard.

**Figure 3:**
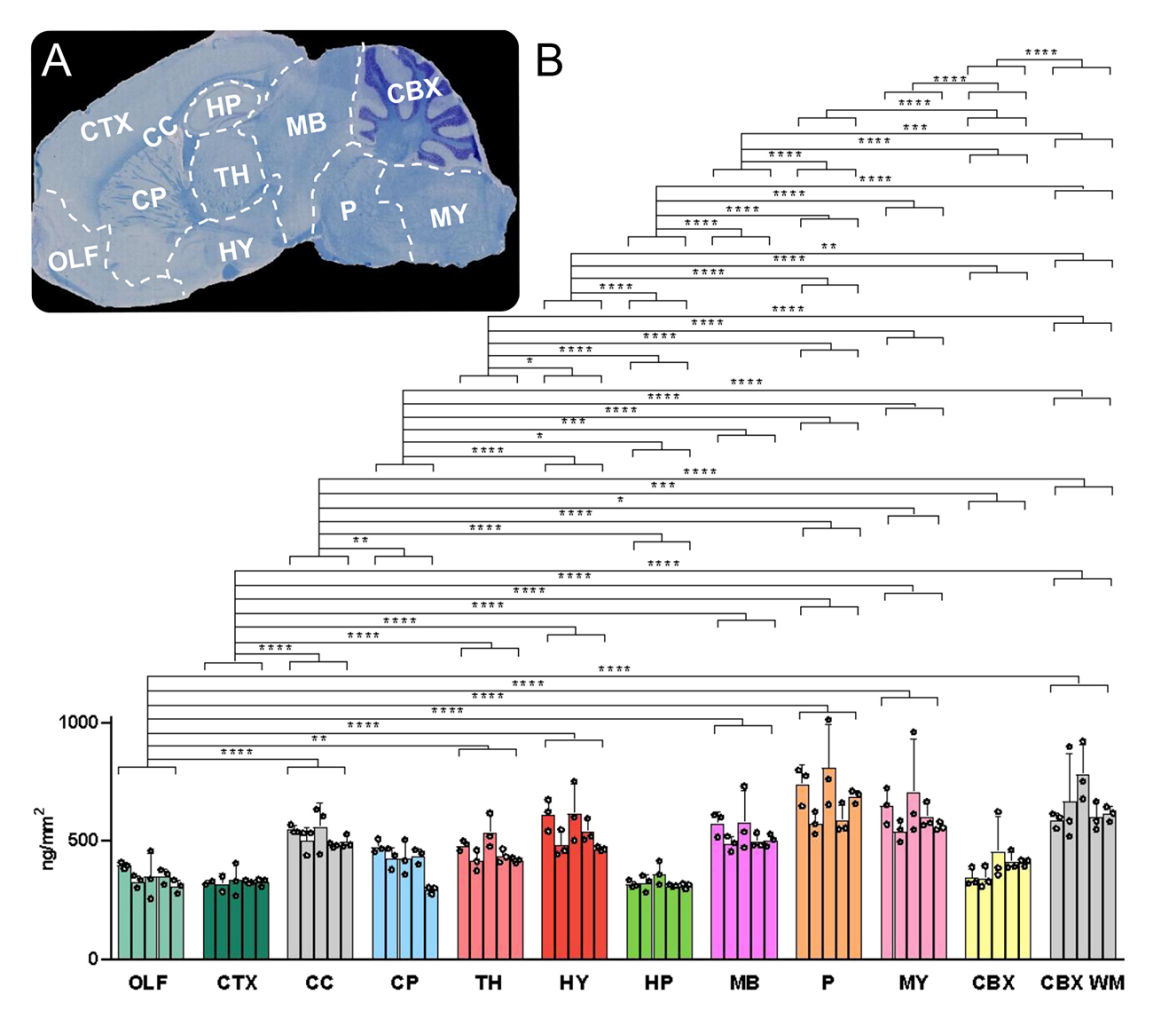
Quantitation of cholesterol in WT adult mouse brain via MSI. (A) LFB/CV staining for myelin of a sagittal mouse brain section adjacent to a section undergoing MSI. Major anatomical structures were identified by comparison with the AMBA^29^ and are outlined with dashed lines: olfactory traits, OLF; cortex, CTX; corpus callosum, CC; caudate-putamen, CP; thalamus, TH; hypothalamus, HY; hippocampus, HP; midbrain, MB; pons, P; medulla, MY; cerebellum, CBX; and cerebellar white matter, CBX WM. (B) Areal density (ng/mm^2^) of cholesterol in brain regions from five WT mice, averaged over different slices (see the Statistics section). Values for individual mice are given by separate histogram bars. The three dots within each bar correspond to region averages for each brain slice employed. The height of each bar represents the mean of the region average for each mouse across the three slices. The error bars indicate the SD of all the sections per mouse. Data were acquired on a vacuum-MALDI-TOF MS instrument.

Using MS^1^, areal densities were determined against a known density of the sprayed-on internal standard in WT mouse brain sections, for selected brain structures (Figure 3A). The linearity of the on-tissue response of endogenous cholesterol versus the sprayed-on deuterated standard was determined by spraying eight consecutive tissue sections with [^2^H_7_]cholesterol at varying known densities (Supporting Information, Figure S2A). Examples of calibration curves obtained on whole-brain sections and considering the cerebellum as an ROI are shown in Supporting Information, Figure S2B,C, respectively. *R*^2^ for the whole brain was determined to be 0.94, and for the cerebellum, it was determined to be 0.97. Our quantitative data reported in Figure 3B and in Supporting Information, Table S2 (ng/mm^2^, mean of five biological replicates ± standard deviation) indicate that cholesterol abundance is the highest in the pons (681.6 ± 123.9 ng/mm^2^) and cerebellar white matter (652.0 ± 119.8 ng/mm^2^) and the lowest in the olfactory traits, cortex, and hippocampus (348.5 ± 52.4, 327.8 ± 32.5, and 326.3 ± 31.6 ng/mm^2^, respectively). Note that cholesterol was quantified via MSI in the whole cerebellum and separately in its WM tracts, whereas cholesterol content in cerebellar grey matter (GM) is estimated from the difference of the total and WM. In previous reports,^11^ cholesterol synthesis and concentration were found to be higher in regions of the central nervous system (CNS) containing heavily myelinated fiber tracts such as the brain stem (medulla and pons) and the midbrain.

In an early report,^8^ it was shown that the concentration of cholesterol in the pons is ∼2.5 times more than in the cortex, which is also in agreement with our data showing a ratio of 2.1. In earlier MSI studies, cholesterol was visualized in mouse brain in coronal or horizontal sections.^18,20,32^ Sagittal MS images have the advantage that the brain stem can be easily differentiated into the midbrain, pons, and medulla regions (Figures 2E and 3A). These brain stem structures show high cholesterol content (Figure 3 and Supporting Information, Table S2), in agreement with previous GC–MS and LC–MS studies.^8,11^

Interestingly, the distribution of gene transcripts of late-stage cholesterol biosynthetic enzymes matches regions of high cholesterol abundance, that is, midbrain, medulla, and pons regions. Please see mRNA expression data of Dhcr24, entrez ID (EID) 74754; Dhcr7, EID 13360; and Sc5d, EID 235293, provided by the AMBA.^29^ Of note, the abundance of cholesterol in the corpus callosum and in the fiber tracts of the caudate-putamen mirrors the distribution of transcripts unique to myelinating oligodendrocytes. See Mbp, EID 17196; Plp1, EID 18823; and Cnp, EID 12799 provided by the AMBA.^29^

**Figure 4:**
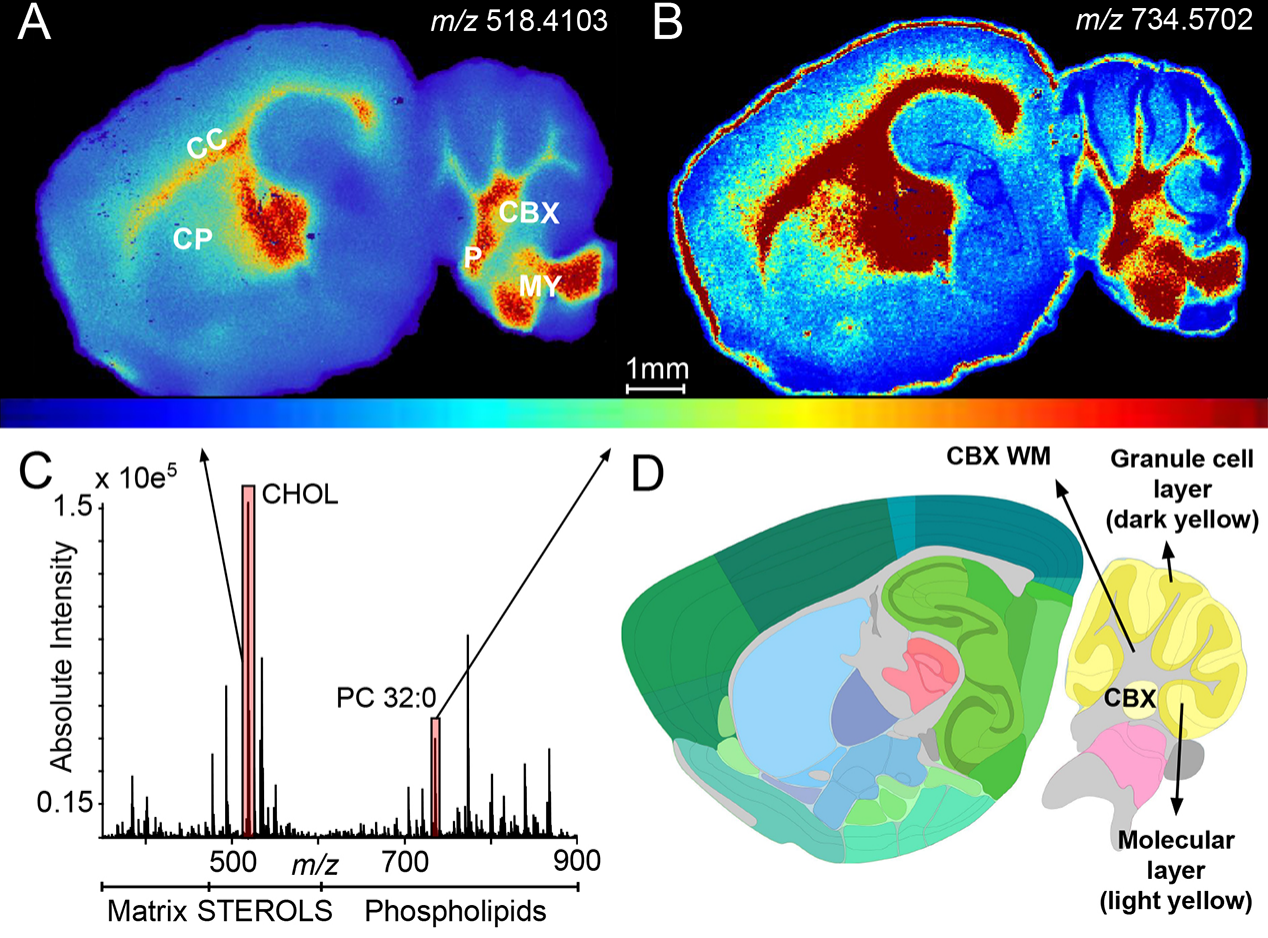
AP-MALDI-MSI of cholesterol in sagittal sections of WT adult mouse brain after on-tissue EADSA. The data were obtained on an Orbitrap instrument. (A) MSI of cholesterol. (B) MSI of PC 32:0. (C) Typical AP-MALDI-Orbitrap MS spectrum averaged over the entire MSI data set showing signals of sterol and other brain lipids that can be detected simultaneously. (D) Anatomical layering of the cerebellum (CBX, yellow). Image from AMBA: Adult Mouse, P56, Sagittal, Image 7 of 21 id = 100883846.^29^ In (A, B), the isolation window width was 7 mmu and the pixel size was 30 μm. Images normalized against sprayed-on [^2^H_7_]cholesterol.

When MS^1^ data obtained by AP-MALDI are visualized in a peripheral sagittal section taken at a plane about 3 mm from the midline, the distribution of GP-derivatized cholesterol at *m*/*z* 518.4103 is clearly enhanced in specific regions of the brain (Figure 4A). These are either WM tracts such as the corpus callosum and cerebellum or brain regions (deep GM structures) containing myelinated fibers, such as the pons, medulla of the brain stem, and caudate-putamen of the diencephalon. In Figure 4, the selected sagittal plane does not include the midbrain but shows hippocampal features.

Notably, using our method for on-tissue cholesterol derivatization and in contrast to ToF-SIMS, other lipids can be mapped simultaneously, particularly when experiments are carried out with API (i.e., AP-MALDI—Orbitrap and DESI—Q-TOF). To show the potential of our approach, we report the MS image of PC 32:0 at *m*/*z* 734.5702 (about 1 ppm deviation from the theoretical *m*/*z*), normalized to [^2^H_7_]cholesterol sprayed-on standard (Figure 4B), but many other peaks could be similarly imaged. Note that the peak at *m*/*z* 734.5702 could also be assigned to PE 35:0. However, phospholipids containing fatty acids with an odd number of carbon atoms are minor species in animals.

Interestingly, in Figure 4A, a continuous gradient of cholesterol concentration is observed going out from the corpus callosum, where cholesterol is at an areal density of about 520 ng/mm^2^, decreasing on moving through the overlying layers of the neocortex. The continuous cholesterol gradient is mirrored by the distribution of PC 32:0 in the same brain regions (Figure 4B). Within the cerebellum, a decreasing concentration of cholesterol is observed going from the WM of the cerebellum (CBX WM, 652.0 ± 119.8 ng/mm^2^) to the granule cell layer and molecular layer of the GM (Figure 4D shows reference anatomy). Note that cholesterol density in cerebellar GM can be estimated (CBX—CBX WM = CBX GM) to be about 260 ng/mm^2^. Here, the cholesterol smooth gradient contrasts with the step gradient shown by PC 32:0 (Figure 4B) which is deficient in the granule cell layer of the cerebellum but more evident in the molecular layer. Further description of MSI of cholesterol in cortical layers and hippocampus can be found in Supporting Information, Figure S3.

The EADSA-MALDI-MSI quantitative assessment of cholesterol areal density in defined brain regions of the WT mouse can be compared with measurements previously obtained with a similar but different experimental approach exploiting low-spatial-resolution (400 μm pixel size) liquid extraction for surface analysis (LESA), that is, EADSA-LESA-LC-MS.^30^Supporting Information, Table S2 reports the values obtained and their standard deviations, in each defined brain region of WT mouse, for both the present and the EADSA-LESA-LC-MS study. The agreement was >90% for very homogenous regions such as the cortex and thalamus, it was about 80% for caudate-putamen, hippocampus, pons, and cerebellar white matter, and it was >67% for heterogeneous midbrain and medulla.

### MSI of Cholesterol in the Developing Mouse

To illustrate how our EADSA-MSI method can be used to monitor brain cholesterol distribution during development, we compared tissues from mice at 1 day and 10 weeks. At birth, myelination is in its very early stage, while at 10 weeks, it is nearly completed.^33^ During development, the cholesterol content in the whole brain goes from about 4 mg/g at birth up to about 15 mg/g in the adult at 26 weeks^34^ and comes from local synthesis only.^1^ During the first 3 weeks of life, when myelin sheaths are being generated, the rates of cholesterol synthesis and accumulation in brain are high at about 250 μg/day^11^ and drop rapidly beyond 3 weeks of age.^34^ Most strains reach sexual maturity between 6 and 8 weeks, so postnatal week 10 can be defined as a young adult mouse.^35^

**Figure 5:**
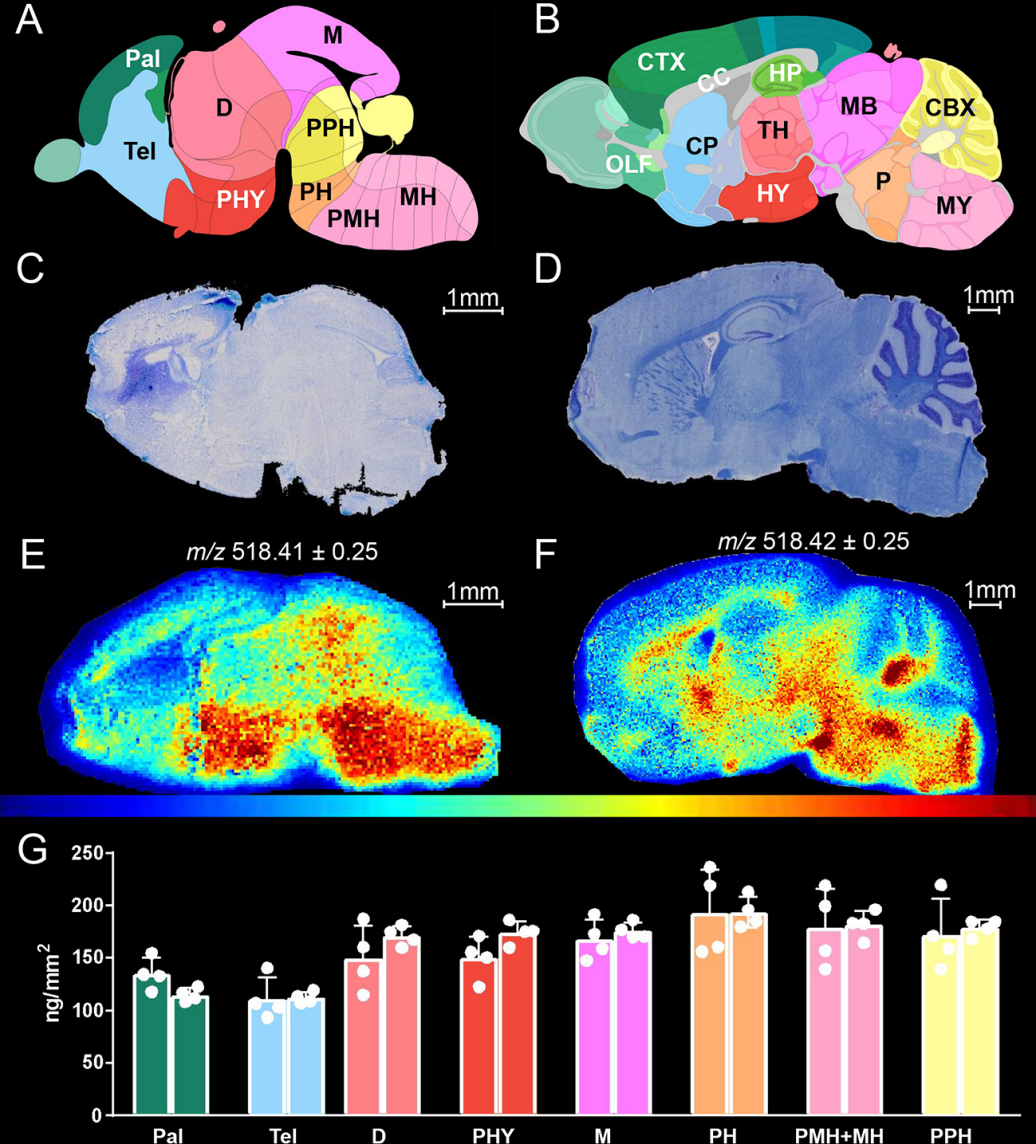
Quantitative MSI of cholesterol in mouse brain at birth and at 10 weeks. In (A,C,E), 1 day-old newborn, and in (B,D,F), 10 week-old adult mouse. (A,B) AMBA images depicting mouse brain sagittal sections with annotations of anatomical structures. The 1 day-old newborn is matched with the E18.5-day embryo atlas image (A). Pallium, Pal; telencephalic vesicle, Tel; diencephalon, D; peduncular hypothalamus, PHY; midbrain, M; pontine hindbrain, PH; pontomedullary and medullary hindbrain, PMH + MH; prepontine hindbrain, PPH. (B) Adult mouse, abbreviations as in Figure 3. (C,D) LFB/CV staining of sagittal mouse brain sections adjacent to sections undergoing MSI. (E,F) MSI of cholesterol after on-tissue EADSA by vacuum-MALDI-TOF MS, normalized against sprayed-on [^2^H_7_]cholesterol, at a pixel size of 50 μm. (G) Areal density (ng/mm^2^) of cholesterol in brain regions from two newborn WT mice, each averaged over four slices. The mean of the region average for individual mice is given by separate histogram bars, and region averages in each slice are represented by dots. The error bars indicate SD. Image from AMBA: Developing Mouse, E18.5, Image 16 of 19 id = 100740373 and Adult Mouse, P56, Sagittal, Image 15 of 21 id = 100883867.^29^

EADSA-MSI was employed to visualize cholesterol distribution in the mouse brain at 1 day and at 10 weeks (Figure 5). We compared MSI of cholesterol with LFB chemical stain and CV as a counterstaining, histological stain for myelin,^27^ as shown in Figure 5C (newborn) and Figure 5D (10 weeks). Figure 5E shows the MSI of cholesterol at 1 day around the time when oligodendrocytes start to contribute to cholesterol synthesis,^36^ and Figure 5F shows the distribution at 10 weeks.

As measured by quantitative EADSA-MSI, the newborn (Figure 5G and Supporting Information, Table S3) shows the highest cholesterol level in the pontine hindbrain (193.4 ± 28.4 ng/mm^2^) and in the medullary and pontomedullary hindbrain (180.3 ± 25.6 ng/mm^2^) that will develop into the cholesterol-rich pons and medulla of the adult mouse (Figure 3B).^33^ The lowest levels of cholesterol are detected in the telencephalic vesicle (111.7 ± 13.9 ng/mm^2^) and in the pallium (125.1 ± 15.3 ng/mm^2^) which will develop into the cortex, olfactory tracts, hippocampus, and caudate-putamen.^33^ Similar to the newborn, these are regions with low cholesterol in the adult (Figure 3 and Supporting Information, Table S2) except for the caudate-putamen which contains some fiber tracts in the adult that are not yet formed in the newborn.^33^ The pro-hypothalamic region (peduncular hypothalamus), which begets the adult hypothalamus and associated fiber tracts, shows a diffused enrichment in cholesterol in the newborn (Figure 5A,E), while the hypothalamus in the adult accumulates cholesterol only in surrounding fibers (Figure 5B,F). A striking difference between 1 day and 10 week animals is the lack of a visible corpus callosum (CC) in the newborn. In the mouse, myelination of the CC is reported to begin at 11 days after birth^37^ and CC is detected by histological methods at around 16–17 days of age.^37^ In contrast to the newborn, in the 10 week adult, the CC is fully formed.^35^ In particular, in the adult, the thicker regions of the CC show enrichment in cholesterol, namely, the rostrum-genu (frontal, 504.3 ± 57.7 ng/mm^2^, see Supporting Information, Figure S4), the body (central, 546.2 ± 66.3 ng/mm^2^), and the splenium (back, 500.4 ± 35.3 ng/mm^2^), while the thinnest part of the CC which is the isthmus connecting the body and the splenium is the CC structure with the lowest cholesterol abundance (457.8 ± 51.2 ng/mm^2^).

Worthy of note, the nonspecific LFB myelin stain of the newborn provides little distributional information when compared to the MSI heat map for cholesterol (Figure 5C cf 5E). Importantly, MSI, as applied here, is specific for cholesterol, while the exact molecular species bound by LFB remain uncertain.^38^ Notably, the cholesterol distribution in the newborn mouse imaged by vacuum MALDI-TOF (Figure 5E) is consistent with the image of an adjacent brain section produced by DESI-Q-TOF (Supporting Information, Figure S5E), proving the robustness of the EADSA-MSI approach.

Finally, as measured by EADSA-MSI, the whole-brain areal density of cholesterol in the newborn is about 160 ng/mm^2^, while it is about 480 ng/mm^2^ in the adult at 10 weeks, showing a threefold increase. Our data are in good agreement with previous reports^11^ where the cholesterol content in the newborn was determined to be about 4 mg/g at birth and to increase to about 10 mg/g at 10 weeks, showing a 2.5-fold increase. In summary, the present data demonstrate that EADSA-MSI can be used effectively to monitor cholesterol abundance in brain structures during development.

### MSI of Cholesterol in the Niemann-Pick Disease Type C1 Shows a Lack of Cholesterol in Hypomyelinated Fibers Tracts

Niemann-Pick disease, type C is a neurodegenerative, lysosomal storage disorder, characterized by accumulation of unesterified cholesterol and sphingolipids in the endo-lysosomal system.^39^ The disease is caused by mutations in the encoding region of genes either for the lysosomal transmembrane protein, NPC1 (95% of cases), or the cholesterol-binding soluble glycoprotein, NPC2 (∼4% of cases).^39^ These two proteins work together to transport cholesterol through the late endosomal–lysosomal membrane into the metabolically active cholesterol pool. Patients with NPC disease show extensive hypomyelination that manifest in cerebral and cerebellar atrophy and WM hypoplasia.^40^

In the present study, we analyzed the cholesterol content and distribution in the brain of the *Npc1*^–/–^ mouse.^41^ In the brain of this mouse, at the 7 week time point, cellular dysfunction translates into loss of many large neurons. Purkinje cells of the cerebellum are particularly sensitive to NPC pathology and are largely lost in patients^42^ and in the mouse model.^43^ Moreover, the brain of the *Npc1*^*–/–*^ mouse generally shows severe dysmyelination of fiber tracts with impairment of oligodendrocyte maturation.^36^ As in patients,^40^ oligodendrocyte loss and dysmyelination may result in hypoplasia of the corpus callosum in this mouse model.^44^ When NPC1/2 proteins are lacking, cholesterol and other lipids remain in the late endosomes/lysosomes and are not transported into the endoplasmic reticulum (ER) and, therefore, sterol homeostasis is undermined by the lack of feedback regulatory mechanisms, that is, free cholesterol accumulates in the late endosome/lysosome compartment, while the rest of the cell perceives a shortage of sterol. Recently, one MSI study has assessed lipid changes in this *Npc1*^*–/–*^ mouse but was limited to the cerebellum.^18^

**Figure 6:**
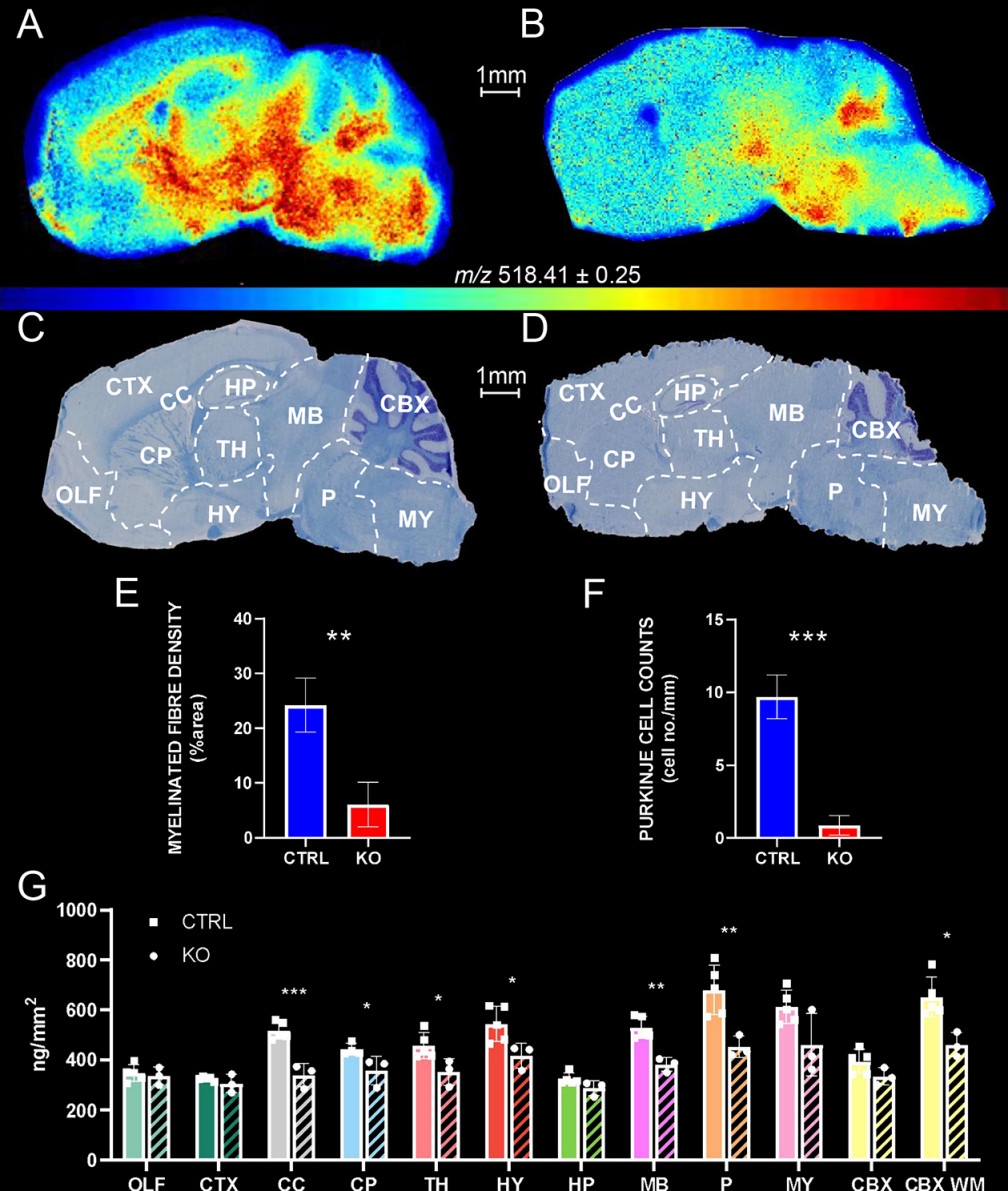
Histology-matched quantitative MSI displaying cholesterol distribution and quantification in brain tissue sagittal sections of WT and *Npc1*^–/–^ mice. (A,B) MSI of cholesterol in WT (A) and in *Npc1*^–/–^ (B) mouse brain tissue. The cholesterol signal was normalized to the signal of sprayed-on [^2^H_7_]cholesterol. Data acquired using vacuum-MALDI-TOF MS at 50 μm pixel size. (C,D) LFB/CV staining of sections adjacent to MSI. Major anatomical structures were identified by comparison with the AMBA^31^ and are outlined with dashed lines. Abbreviations are the same as in Figure 3. (E) Myelinated fiber density in the CP and (F) Purkinje cell counts in the CBX of WT and of *Npc1*^–/–^ mice, as assessed by quantitative morphometry of stained sections. (G) Areal density (ng/mm^2^) of cholesterol in brain regions from five WT and three *Npc1*^–/–^ mice. Group averages are given by separate histogram bars. Dots within each bar correspond to the individual mouse average. (■CTRL or •KO). The error bars indicate the SD. Data were acquired on a vacuum-MALDI-TOF MS instrument.

In the present study, we have exploited MSI to study the whole brain. The chosen time point was of 10 weeks when the phenotype is severe but not yet lethal: *Npc1*^*–/–*^ mice die around 12 weeks of age.^45^Figure 6A,B shows the MSI heat maps of cholesterol distribution in WT and *Npc1*^–/–^ mouse brain, respectively. These heat maps can be compared with Figure 6C,D, showing LFB/CV staining for myelin of the corresponding adjacent brain tissue sections. For further comparison of MSI with histology, the density of myelinated fibers in the caudate-putamen (Figure 6E and Supporting Information S6A) and the number of Purkinje cells in the cerebellum (Figure 6F and Supporting Information S6B) of WT and *Npc1*^–/–^ mouse were determined from the histological data obtained via LFB/CV staining. Figure 6G shows cholesterol levels in selected brain ROI, as quantified by MSI for the *Npc1*^*–/–*^ and WT mice. The quantitative data reported in Figure 6G and in Supporting Information, Table S2 indicate that in *Npc1*^–/–^ brain, cholesterol is most abundant in the cerebellar white matter, medulla, and pons (462.1 ± 58.7, 461.6 ± 111.3, and 454.2 ± 49.5 ng/mm^2^, respectively) and least abundant in the cortex and hippocampus (307.4 ± 36.9 and 288.9 ± 36.5 ng/mm^2^, respectively).

Supporting Information, Table S2 also reports the % difference in cholesterol areal density between the *Npc1*^–/–^ and WT animals. The regions showing the highest reduction of cholesterol in the *Npc1*^–/–^ mouse compared to the WT are the corpus callosum (34.4%), pons (33.4%), cerebellar WM (29.1%), and midbrain (27.8%) (*p*-values are indicated in Figure 6G).

A comparison of histological and MSI data in WT and *Npc1*^–/–^ mouse brain reveals structural differences that can be correlated with compositional changes in cholesterol distribution and abundance. The most striking difference is in the CC.

Figure 6C shows that the CC in the WT mouse is heavily myelinated and highlighted by the LFB dye. In contrast, Figure 6D shows that the CC is apparently nonmyelinated in the *Npc1*^–/–^ brain with the LFB stain showing this structure as mostly white. This correlates well with our MSI data where cholesterol areal density is significantly higher in the WT as compared to the *Npc1*^–/–^ CC (Figure 6G, ***p-value < 0.001).

Other than in the CC, the significantly higher cholesterol areal density as determined by MSI in the caudate-putamen region and in the cerebellar WM of the WT mouse as compared to the *Npc1*^–/–^ mouse (*p-values < 0.05, Figure 6G) also relates to known histological markers.^44^ This prompted us to further analyze histological data by assessing the percentage of myelinated fibers in the caudate-putamen (Figure 6E and Supporting Information S6A) and the number of Purkinje cells in the cerebellar GM (Figure 6F and Supporting Information S6B) of these mice. In the caudate-putamen, myelinated fiber density assessed in LFB/CV-stained sections was found to be significantly reduced in the *Npc1*^–/–^ mouse as compared to WT (Figure 6E and Supporting Information S6A, **p-value < 0.01), agreeing with MSI measurement of cholesterol areal density in the same brain region (Figure 6G, *p-value < 0.05).

Focusing on the cerebellum, the MSI measurements show a significant difference when cerebellar WM is considered (Figure 6G, *p-value < 0.05). This is in agreement with a previous MSI study,^18^ similarly showing a reduced cholesterol signal intensity, as normalized by the total ion current (TIC), in the cerebellum of the same *Npc1*^–/–^ mouse compared to WT. Interestingly, there is significant reduction of the number of Purkinje cells in the GM of the *Npc1*^*–/–*^ cerebellum (Figure 6F and Supporting Information S6B, ***p-value < 0.001). Loss of Purkinje cells is a known phenotypic marker of Niemann-Pick type C patients^42^ and animal models.^43^ These neuronal cells have their cell bodies residing in the cerebellar GM, but their myelinated axons establish postsynaptic connections with cerebellar deep nuclei in the WM.^46^ Therefore, a reduction in the cholesterol content of the cerebellar WM of the *Npc1*^–/–^ mouse could be explained in part by the loss of Purkinje cell efferent and afferent connections.

A significant reduction in the cholesterol areal density of the hypothalamus, midbrain, and pons in the *Npc1*^–/–^ mouse compared to WT was revealed by MSI (Figure 6G, *p-value < 0.05 and ** *p*-value < 0.01). Histological staining shown in Figure 6C,D illustrates reduced LFB stain density in these same areas of the *Npc1*^–/–^ mouse, correlating with MSI heat maps of cholesterol in Figure 6A,B, respectively.

### EADSA-MSI with Multiple Ionization Modes and Analyzers

We assessed the robustness of our EADSA-MSI method on different mass spectrometers having different sources (vacuum-MALDI, AP-MALDI, and DESI) and analyzers (Q-TOF with and without ion mobility, linear TOF, and Orbitrap). Our data are consistent as shown for the cholesterol distribution in adult mouse in Figures 1C, 5F, 6A, and Supporting Information S2A all generated by vacuum-MALDI-TOF, in Supporting Information Figure S5A generated by MALDI-Q-IM-TOF, in Supporting Information Figure S5C generated by DESI-Q-TOF, and in Figure 4A generated by AP-MALDI-Orbitrap. Altogether, these data illustrate the reproducibility and robustness of our EADSA-MSI methodology.

## Conclusions and Perspective

The EADSA-MSI method presented provides a tool for the quantitative imaging of cholesterol in mouse brain tissue sections. On-tissue EADSA was successfully employed to improve the analytical power of MSI toward sterols, allowing quantitative mapping of cholesterol at pixel sizes down to 30 μm. Different MS platforms were utilized including vacuum-MALDI-TOF, vacuum-MALDI-Q-IM-TOF, AP-MALDI-Orbitrap, and DESI-Q-TOF, demonstrating the robustness of the method toward different ionization sources, analyzers, and detectors. With atmospheric pressure ionization-MSI (AP-MALDI-Orbitrap and DESI-Q-TOF), the method allowed detection of other lipid classes (phospholipids) in parallel to derivatized sterols, thereby extending the reach of the methodology to the characterization of diverse lipid markers simultaneously. Experiments are underway to extend the reach of the methodology to less abundant and isomeric sterols, including oxysterols, by integrating MS^3^ analyses in the imaging workflow. MSI is a rapidly advancing technology that can reach cellular resolution,^47^ thereby providing information on changes happening both at the structural and cellular level. Bridging MS-based lipidomics with histopathology will allow the correlation of quantitative molecular information with the anatomical location, opening a further window for the entry of MSI into clinical chemistry. The EADSA-MSI method described here for imaging of cholesterol directly on tissue can be easily applied to a number of scientific fields including neuroscience, pharmacology, biochemistry, and pathology. Particularly, its application to the study of diseases such as Alzheimer’s and multiple sclerosis has the potential to unveil the role of cholesterol in these important neuropathologies.

## Supporting Information Available

Experimental details on animal models; histology; stereology; calculation of areal densities; MSI file size and computational resources used; MS^3^ fragmentation patterns; calibration curves; high-resolution capability as a measure of homogeneity of the sprayed-on isotope-labeled standard; robustness; quantitative morphometry; comparison of normalization strategies; tables relating MSI instruments to figures; comparison of MALDI and LESA data; and cholesterol areal densities in developing mouse brain (PDF)
