# Supplementary material for: Visualizing Cholesterol in the Brain by On-Tissue Derivatization and Quantitative Mass Spectrometry Imaging
Source: Anal Chem. 2021 Mar 9;93(11):4932–43. doi: 10.1021/acs.analchem.0c05399 (PMC7992047; doi:10.1021/acs.analchem.0c05399)
Supplement: Supplementary file 1 — ac0c05399_si_001.pdf [file ac0c05399_si_001.pdf]

SUPPORTING INFORMATION

Visualising Cholesterol in the Brain by On-Tissue Derivatisation and Quantitative Mass Spectrometry Imaging

Roberto Angelini[1], Eylan Yutuc[1], Mark F Wyatt[1], Jillian Newton[2], Fowzi A Yusuf[1], Lauren Griffiths[1], Benjamin J Cooze[1], Dana El Assad[3], Gilles Frache[3], Wei Rao[4], Luke Allen[5], Zeljka Korade[5], Thu TA Nguyen[6], Rathnayake AC Rathnayake[6], Stephanie M Cologna[6], Owain W Howell[1], Malcolm R Clench[2], Yuqin Wang[1], William J Griffiths[1*].

[1]Swansea University Medical School, Singleton Park, Swansea, SA2 8PP, Wales, UK.

[2]Centre for Mass Spectrometry Imaging, Biomolecular Research Centre, Sheffield Hallam University, Howard Street, Sheffield, S1 1WB, UK.

[3]Luxembourg Institute of Science and Technology, Materials Research and Technology, L-4422 Belvaux, Luxembourg.

[4]Waters Corporation, European Application Laboratory, Stamford Avenue, Altrincham Road, Wilmslow, SK9 4AX, UK.

[5]Departments of Pediatrics and Biochemistry and Molecular Biology, University of Nebraska Medical Center, Omaha, NE 68198, USA.

[6]Department of Chemistry and Laboratory of Integrated Neuroscience, University of Illinois at Chicago, Chicago, IL 60607, USA.

* Corresponding author: William J Griffiths

Email: w.j.griffiths@swansea.ac.uk

ORCIDs: RA, 0000-0001-5136-5921; EY, 0000-0001-9971-1950; MFW, 0000-0003-4107-5941; JN, 0000-0003-4848-7391; DEA, 0000-0001-7454-4236; GF, 0000-0003-0069-9475; LBA, 0000-0002-7043-3376; ZK, 0000-0002-8690-4507; TTAN, 0000-0001-8617-3305; RACR, 0000-0001-8115-2812; SMC, 0000-0002-3541-3361; OWH, 0000-0003-2157-9157; MRC, 0000-0002-0798-831X; YW, 0000-0002-3063-3066; WJG, 0000-0002-4129-6616.

Keywords

Mass Spectrometry Imaging; Quantification; MALDI; Brain; Derivatisation; Cholesterol; Myelin, Sterol; Development; Niemann-Pick disease.

Table of Contents

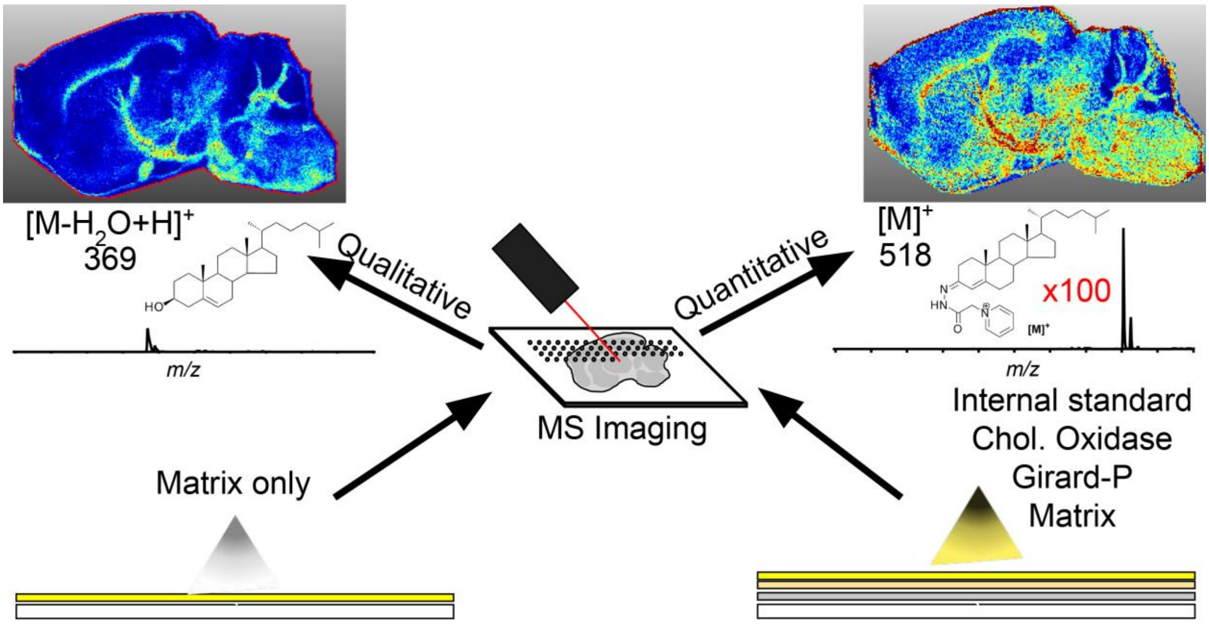

Supplemental Methods

The aim of the study was to develop an MSI method suitable to map the distribution and to determine the concentration of cholesterol in different anatomical regions of the mouse brain.

Experimental models

In the present study WT and *Npc1*$^{-/-}$ mice were employed. We analysed the cholesterol content and distribution in the brain of the *Npc1*$^{-/-}$ mouse where the gene was knocked out by a naturally occurring retroposon-driven frameshift mutation (BALB/cNctr-*Npc1*^{m1N}/J): https://www.jax.org/strain/003092. Genotype was confirmed by PCR as previously reported[1]. At ten weeks of age, male WT (*Npc1*$^{+/+}$) and null (*Npc1*$^{-/-}$) mice were euthanized via CO_2 asphyxiation followed by decapitation. Whole brain was removed, frozen in dry ice and stored at -80°C. All procedures were performed in accordance with the Guide for the Humane Use and Care of Laboratory Animals. Experiments were performed in accordance with University of Illinois at Chicago IACUC approved protocols[1].

To study cholesterol distribution during mouse development, the phenotypically normal *Dhcr7*$^{T93M/+}$ mouse was used[2]. In the present study one-day-old newborn animals (P0) were employed. All mice were housed under a 12-hr light-dark cycle at constant temperature (25 °C) and humidity with *ad libitum* access to food (Teklad LM-485 Mouse/Rat Irradiated Diet 7912) and water at the University of Nebraska Medical Center. Newborn mice were used for the study. All procedures were performed in accordance with the Guide for the Humane Use and Care of Laboratory Animals. The use of mice in this study was approved by the Institutional Animal Care and Use Committee of University of Nebraska Medical Center. Note our initial aim was to also image brain from the newborn *Dhcr7*$^{T93M/\Delta3-5}$ mouse, however, brain from this animal proved particularly difficult to section, and will require further investigation.

Adult mice for exploratory experiments were kindly provided by Dr Rosalind John and brain dissected by Bridget Allen (Cardiff University). Mice were CD1, females, 7 months old. Mice were euthanized by dislocation of the neck (schedule 1) in accordance with institutional animal care guidelines and brains dissected immediately post-mortem and snap-frozen in liquid nitrogen. Mice were housed in a conventional unit on a 12-hr light–dark cycle with lights coming on at 06:00, with a temperature range of 21 ± 2°C, and with free access to tap water and standard chow. All procedures were conducted in accordance with the requirements of the UK Animals (Scientific Procedures) Act 1986, under the remit of Home Office licence (BA) with additional ethical approval at Cardiff University.

Histology

Luxol Fast Blue (LFB) and Cresyl Violet (CV) staining was performed according to Kluver and Barrera[3] on tissue sections adjacent to sections analysed by MSI. Snap frozen sections previously stored at -80°C, were allowed dry at room temperature for 20 min, and then incubated with 4% PFA for 1 hr (~100 µL on each section). Afterwards, the sections were dehydrated in 70% then 95% ethanol, for 2 min each. Subsequently, sections were incubated overnight (< 16 hr) in a sealed jar at 40°C in 0.1% LFB in 95% ethanol. The LFB solution was filtered and preheated before incubation. Afterwards, excess stain was removed by gently washing the sections in a saturated solution of lithium carbonate until WM and GM could be distinguished. The LFB stained slides were counterstained with 0.1% Cresyl Violet acetate solution preheated at 65°C for 30 min and then quickly washed in water and dehydrated in 95% ethanol then absolute ethanol (1-2 seconds each). Afterwards the slides were cleared in xylene overnight and dried at room temperature for a few seconds.

Stereology

Firstly, the mouse brain was sectioned through a sagittal plane. Then stained sagittal sections were matched with images in the corresponding reference atlas. The shape of the fibres tracts as detected by LFB was used to identify the correct atlas reference image having a comparable distance from the midline. Then the LFB/CV stained sections and the MSI heat maps were overlaid together with the appropriate reference atlas images, and anatomical regions identified were outlined with dashed lines defining ROI.

For MALDI-MSI data analysis of the adult mouse, three sagittal sections at about 1.3 mm ± 300 µm distance from the midline were employed. The three chosen sections were taken at sagittal planes separated by 100 µm. For histology data analysis we employed sections adjacent to MSI on the left and on the right for a total of 6 sections per mouse that covered the area 1.3 mm ± 400 µm distant from the midline. The atmospheric pressure (AP)-MALDI data was an exception and obtained on a more peripheral section (about 3 mm from the midline). The mouse brain AMBA images employed as reference were: Adult Mouse, P56, Sagittal, Images 13-16 of 21 ids= 100883818, 100883869, 100883867, 100883888[4].

For MALDI-MSI data analysis of the newborn mouse, four consecutive sagittal sections at about 0.6 mm ± 100 µm from the midline were employed. For histology, two sections adjacent on the far left and on the far right of the series of four used for MSI were employed. The newborn AMBA image employed as reference was Developing Mouse, E18.5, Sagittal, Image 16 of 19 id= 100740373[4].

Calculations of areal densities of isotope-labelled standard, cholesterol oxidase and GP-hydrazine

To calculate areal densities of [$^2H_7$]cholesterol, cholesterol oxidase and GP-hydrazine we employed the following equation:

(1) Areal density (mg/mm^2) = [no. layers x concentration (mg/mL) x flow rate (mL/min)] / [spray speed (mm/min) x line distance (mm)]

[$^2H_7$]Cholesterol in ethanol (200 ng/µL) was sprayed on-tissue using a SunCollect automated pneumatic sprayer at a flow rate of 0.02 mL/min, a spray speed of 900 mm/min, a line distance of 2 mm, for 18 layers. Using (1), the areal density on brain of [$^2H_7$]cholesterol was calculated to be 40 ng/mm^2. The cholesterol oxidase activity was 0.264 U/mL in the sprayed solution, using eq.1 and the same spray parameters as for the internal standards, this translates to an areal density of 0.05 mU/mm^2. The concentration of the chloride salt of GP sprayed on tissue was 5 mg/mL, using the same spray parameters as above this translated to an areal density of 1.00 µg/mm^2 (5.3 nmol/mm^2).

MSI file size and computational resources

Regarding the vacuum-MALDI experiments at 50 µm pixel size (Bruker ultrafleXtreme), acquisition time for one mouse brain tissue sagittal section was typically about 11.5 hr for a total of ~27,000 positions and a file size of about 2 GB. The loading time of such a raw file was about 45 min on a desktop computer having 6 GB RAM, one i7 processor and regular SATA hard drive; while it was about 5 min on a workstation with 64 GB RAM, two 10-Core Processor (13.75Mb Cache, 2,20 GHz) and an NVME SSD hard drive. The image was directly visualized by Flex Imaging 3.0 (Bruker) or analyzed with SCiLS Lab 2014b (SCiLS, Bremen, Germany), in which case the image file produced out of the raw file has an average size of 20 GB (including all annotations and ROI analyses).

For AP-MALDI experiments at 30 µm pixel size (ThermoFisher Orbitrap Elite) the acquisition of one mouse brain tissue sagittal section was achieved in about 15 hr for a total of ~90,000 positions and a file size of about 5 GB. The raw file was then loaded into Image Quest (ThermoFisher) and exported into imzML format in about 2 hr. The imzML image file (centroid) produced out of the raw file has average size of about 0.5 GB, this was then imported into SCiLS Lab MVS 2014c (SCiLS, Bremen, Germany) with a loading time of about 10 min on the workstation with 64 GB RAM, two 10 Core Processor (13.75Mb Cache, 2,20 GHz) and NVME SSD hard drive. Here the imzML file was further converted obtaining the final image file of ~1.5 GB. Data for this study is deposited at https://osf.io/39sj7/.

High spatial resolution of fine hippocampal structures via AP-MALDI-Orbitrap

Shown in Supplemental Figure S8 are high spatial resolution images of hippocampal structures. The pyramidal and granule cell layers are evident in AP-MALDI-Orbitrap MSI and LFB/CV stained images. As seen in the LFB/CV stained section the pyramidal layer is about is about 75 µm thick, about 3-4 large neurons in the y-dimension. This translates to about 3 pixels of 25 µm size in the AP-MALDI-Orbitrap image.

Comparison of normalisation strategies

In Supplemental Figure S9 cholesterol abundance normalised to [$^{2}H_{7}$]cholesterol and to TIC are compared. Both normalisation strategies reveal similar patterns of cholesterol abundance in macro-structures i.e. pons, medulla, corpus callosum and midbrain, however, small subregions of the brain enriched in cholesterol are more clearly visualized in the [$^{2}H_{7}$]cholesterol-normalized image than with TIC normalization.

MSI reference table

To assist reading of the manuscript, Table S1 containing information on instruments used in each figure/experiment is included.

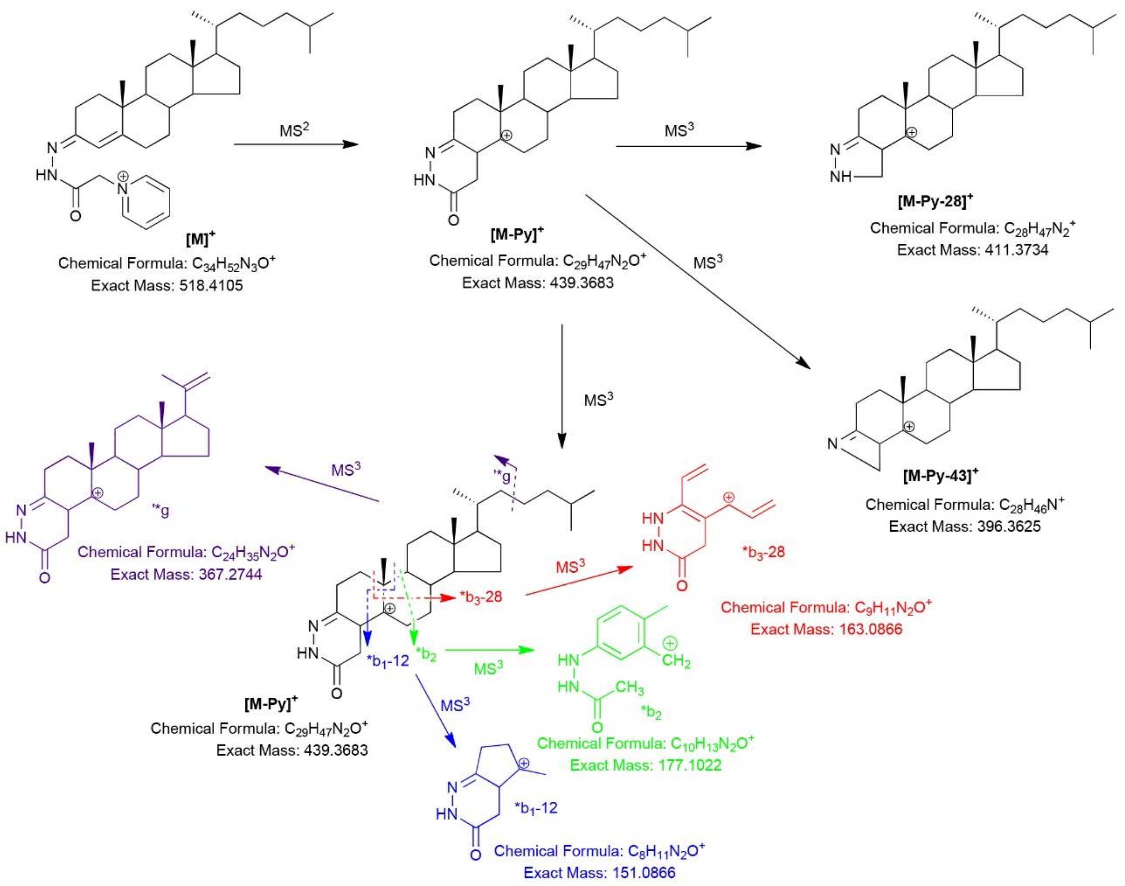

Supplemental Figure S1. Fragmentation pattern of GP-derivatised cholesterol upon MS^n analysis showing the chemical structures of the major diagnostic fragment ions.

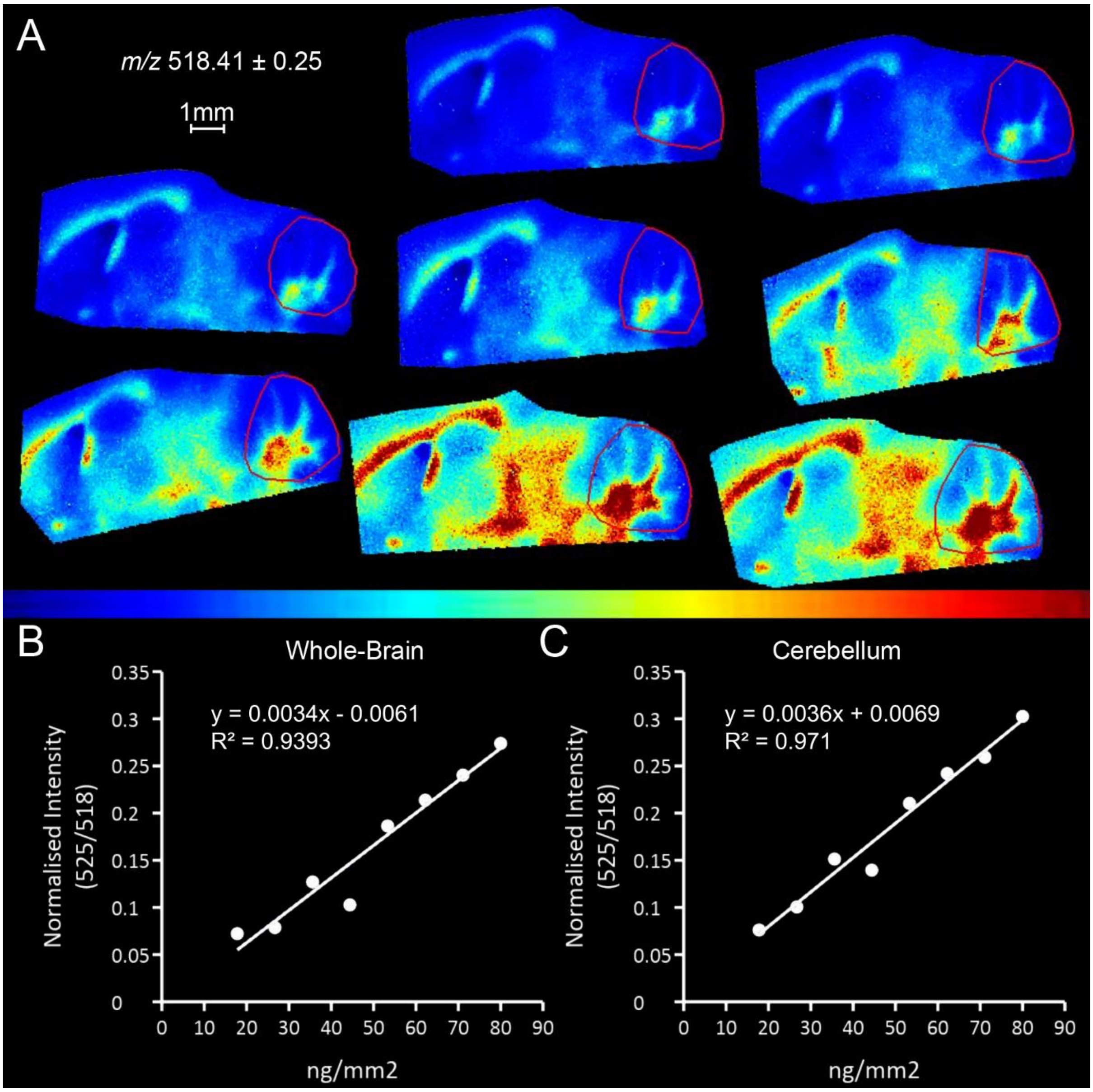

Supplemental Figure S2. Generation of calibration plots for endogenous cholesterol against sprayed-on [$^2H_7$]cholesterol on mouse brain tissue. To determine absolute quantities of cholesterol, calibration curves were constructed by spraying a solution of [$^2H_7$]cholesterol at varying concentrations onto consecutive mouse brain tissue sections. (A) From left to right and top to bottom, MSI depicting cholesterol normalized to the peak of [$^2H_7$]cholesterol sprayed on-tissue at decreasing concentration of [$^2H_7$]cholesterol. (B) Calibration curve generated using the entire section. (C) Calibration curve generated using the cerebellar area outlined in red. Data normalized to sprayed-on [$^2H_7$]cholesterol are shown using a "jet" scale over a single range. Scale bar = 1 mm, spatial resolution 50 µm. Data were acquired on a vacuum-MALDI-TOF MS.

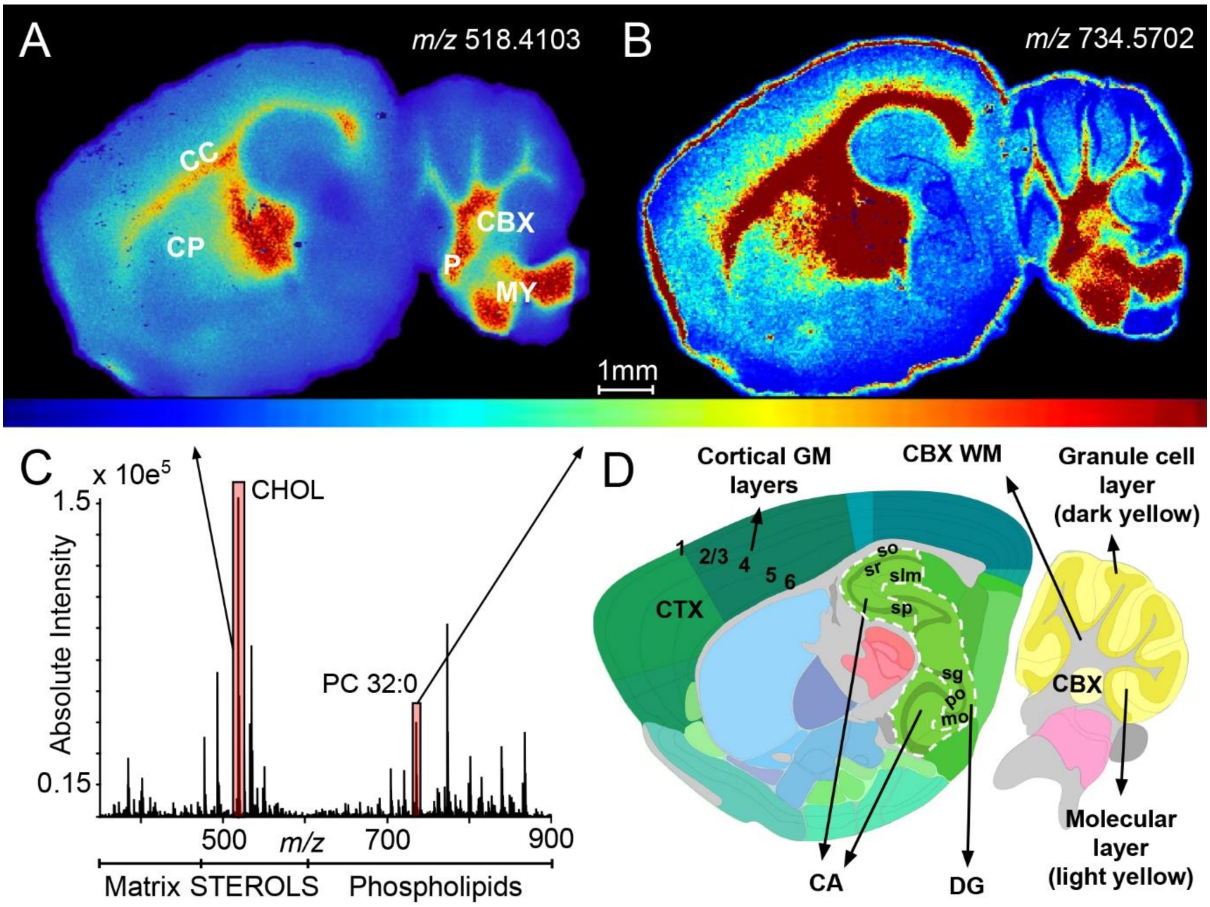

Supplemental Figure S3. AP-MALDI-MSI of cholesterol in sagittal sections of WT adult mouse brain. The data was obtained on Orbitrap instrument. (A) Distributional heat map of cholesterol. (B) Distributional heat map of a major structural phospholipid assigned to PC 32:0. (C) Typical AP-MALDI-Orbitrap spectrum averaged over the entire MSI dataset after on-tissue EADSA derivatization showing sterol and phospholipid (and other brain lipids) signals that can be detected simultaneously. (D) Anatomical layering of cortex, cerebellum and hippocampus. Layers of the cortex (CTX, dark green) and of the cerebellum (CBX, yellow) are shown. Cerebellar WM is in the lobules (coloured grey) of the cerebellum (CBX) which are surrounded by the granule cell layer (dark yellow) and then by the molecular layer (light yellow). Layers of the dorsal and ventral hippocampal formation are also visible in the selected sagittal plane in both Ammon's horn (CA) and Dentate Gyrus (DG). The CA is layered into (dorsal to ventral) strata oriens (so), radiatum (sr) and lacunosum-moleculare (slm) (all in light green) with the pyramidal layer (sp, dark green) stratified between the strata oriens (so) and radiatum (sr). The DG shows the central granule cell layer (sg, dark green) in between the molecular (mo) and polymorph (po) layers (both in light green). Image credit Allen Institute for Brain Science: Adult Mouse, P56, Sagittal, Image 7 of 21 id=100883846[4]. In (A, B) isolation window width was 7 mmu, pixel size was 30 µm, mass deviation for cholesterol was <1 ppm using [$^2H_7$]cholesterol as a lock mass. Images normalized against sprayed-on [$^2H_7$]cholesterol.

Differential gradients for both cholesterol and PC 32:0 can be observed also in the Ammon's horn and the dentate gyrus of the hippocampal formation. Figure S3B shows that PC 32:0 is deficient in the pyramidal layer of the Ammon's horn and in the granule cell layer of the dentate gyrus while it is more evident in the strata oriens, radiatum and lacunosum-moleculare of the Ammon's Horn as well as in the molecular and polymorph layers of the dentate gyrus (Figure S3D shows reference anatomy). The

step gradient of PC 32:0 demonstrates the outlines of the Ammon’s horn and of the dentate gyrus, while the cholesterol distribution gradient is more continuous across these structures (Figure S3A). Indeed cholesterol, although being more concentrated in the myelin sheaths of the WM, is a key component of all membranes and, therefore, is present throughout the brain. Moreover, cholesterol is by far the major representative of its class, with other sterols present at comparatively low abundance and not exerting any known structural function in membranes. On the contrary membrane phospholipids such as PC, have high structural diversity and may show both a continuous gradient (as in the cortex), or a step gradient (as in cerebellum and hippocampus) for an individual molecular species, even within layers of the same brain structure.

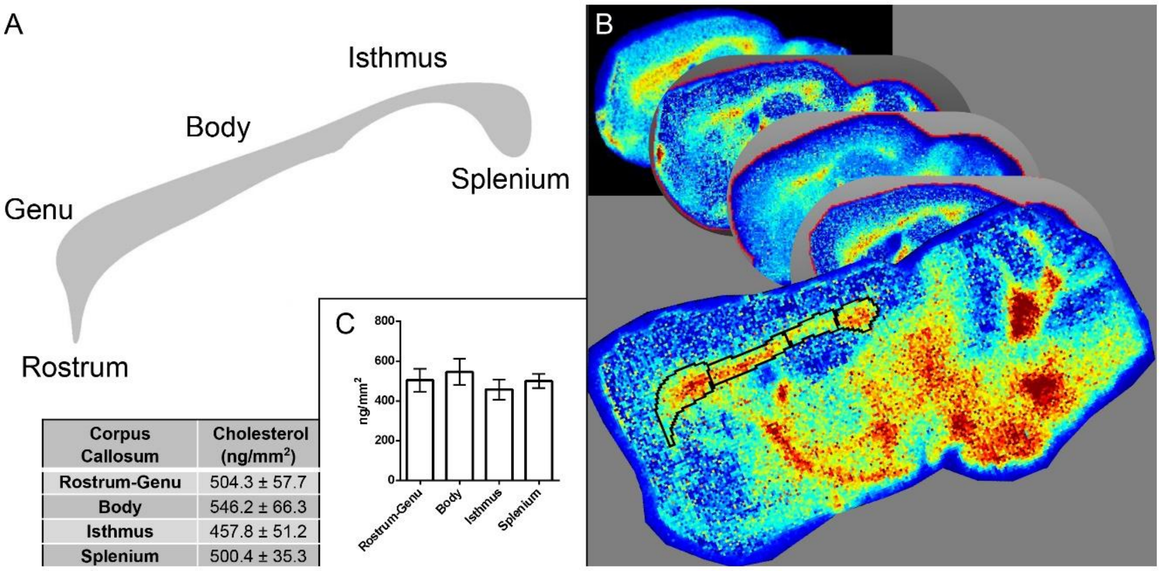

Corpus Callosum	Cholesterol (ng/mm^2)
Rostrum-Genu	504.3 ± 57.7
Body	546.2 ± 66.3
Isthmus	457.8 ± 51.2
Splenium	500.4 ± 35.3

Supplemental Figure S4. (A) Gross anatomy of the corpus callosum. The corpus callosum is approximately 7 mm in length and is C-shaped in a gentle upwardly convex arch. It is divided into four parts (from anterior to posterior): rostrum (continuous with the lamina terminalis), genu, trunk/body, splenium. Body and splenium are connected by the thinnest isthmus. (B) Reproducibility of the morphology of the Corpus Callosum in the five WT mice analysed, one section per mouse is shown. An example of the ROI for CC structure is also shown. (C) Areal density (ng/mm^2) of cholesterol in brain regions from five WT mice, averaged over the sections (n = 5 mice, 3 sections per mouse for a total of 15 measurements). Average values for CC regions in the WT mice group are given by separate histogram bars. The height of each bar represents the mean of the region average in the WT mice group. The error bars indicate the SD of all the replicates (sections) per mouse. Data were acquired on a vacuum-MALDI-TOF MS.

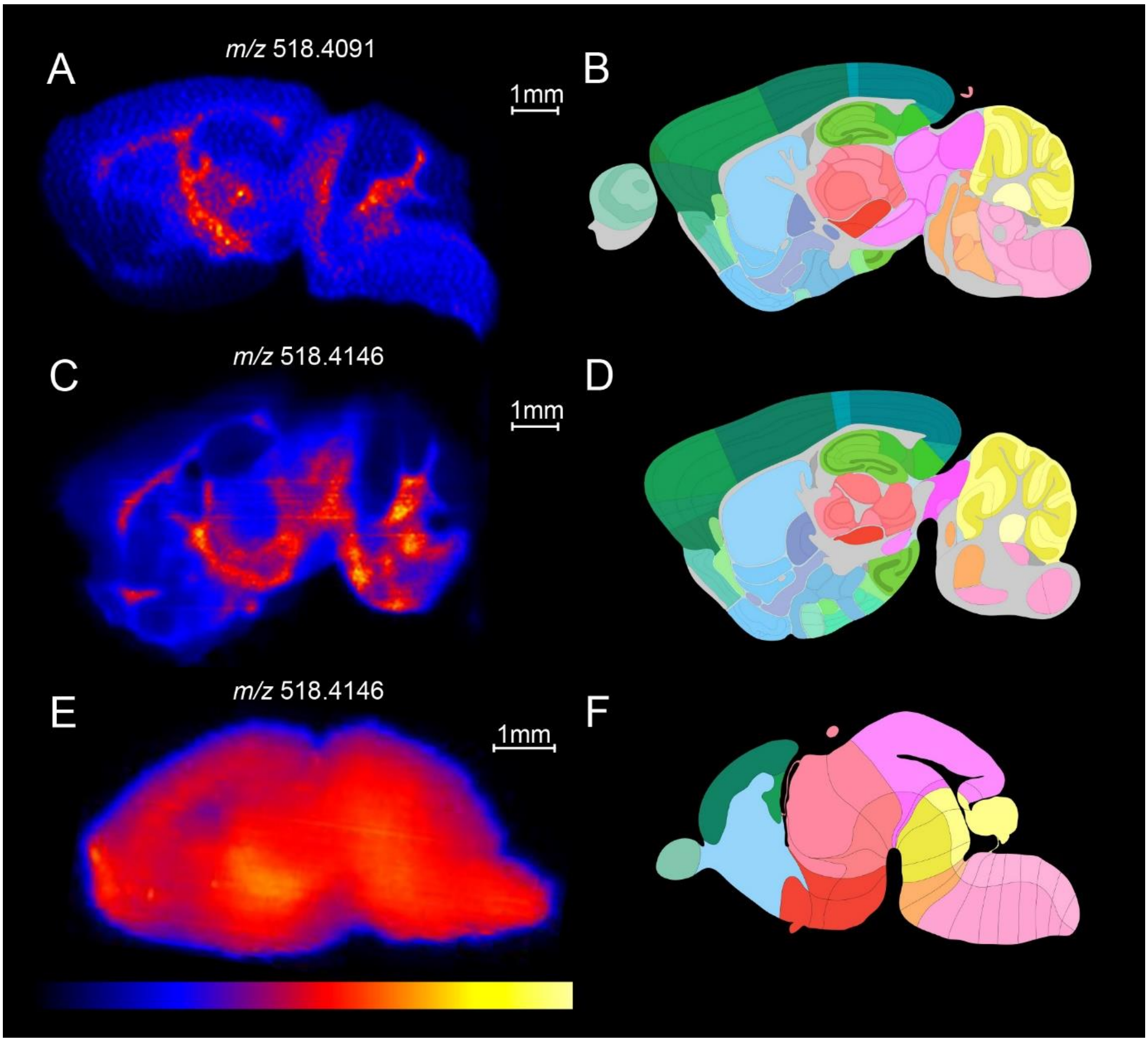

Supplemental Figure S5: MSI depicting cholesterol in WT mouse brain tissue obtained by on-tissue EADSA derivatisation and subsequent MS analysis together with corresponding reference atlas sagittal sections. (A) Vacuum-MALDI-Q-TOF MSI of adult mouse brain cholesterol and (B) reference atlas section corresponding to the same sagittal plane. Image credit Allen Institute: Mouse, P56, Sagittal, Image 11 of 21 id= 100884129[4]. (C) DESI-Q-TOF MSI of cholesterol in adult WT mouse brain and (D) corresponding reference atlas sagittal section. Image credit Allen Institute for Brain Science: Mouse, P56, Sagittal, Image 9 of 21 id= 100883813[4]. (E) DESI-Q-TOF MSI of cholesterol in 1-day-old WT mouse brain and (F) corresponding reference atlas sagittal section. Image credit Allen Institute for Brain Science: Developing Mouse, E18.5, Image 16 of 19 id=100740373[4]. For MSI experiments: isolation window width 7 mmu, pixel size 50 µm (A) and 45 µm (C & E); data analysed by Mass Lynx (Waters) visualised on a BPROY scale. All images data are normalised to sprayed-on [$^{2}H_{7}$]cholesterol. Please note that Supplemental Figures S5A and S5C represent sagittal sections of different WT mice and were taken on different planes separated by about 300 µm. Supplemental Figure S5A is matched with the corresponding reference section from the Allen Mouse Brain Atlas[4] in Figure S5B, and Figure S5C is matched with the appropriate atlas section in Figure S5D.

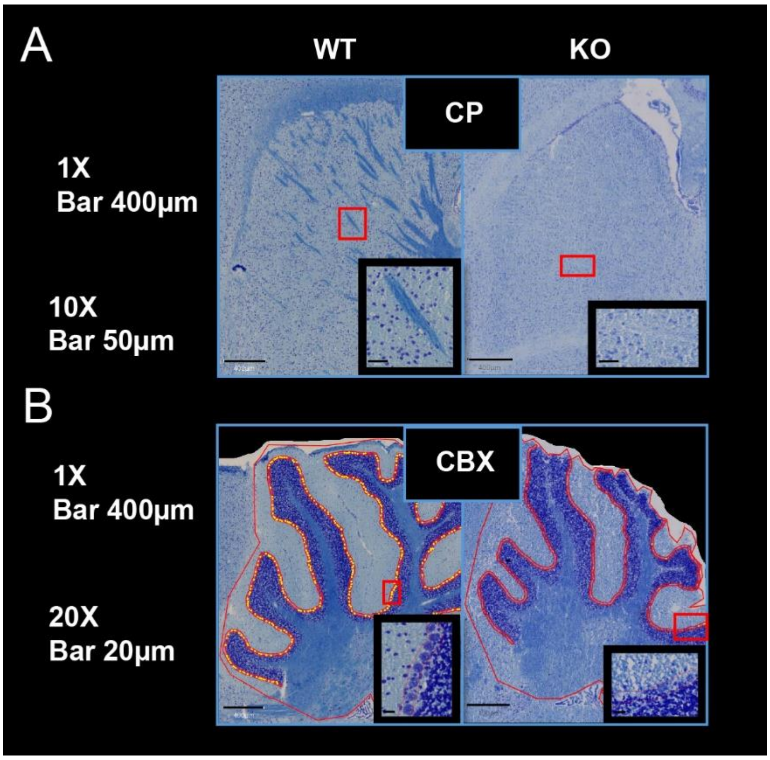

Supplemental Figure S6: LFB/CV histological staining of WT (left panels) and *Npc1*$^{-/-}$ mouse brain (right panels). (A) Brain region of the caudate-putamen showing myelinated fibres in the WT but absent from the *Npc1*$^{-/-}$ mouse. In the enlargements a single fibre can be seen in the WT. (B) Cerebella region showing (with Qu Path annotation for Purkinje cells) the difference between WT and *Npc1*$^{-/-}$ in the number Purkinje cells. In the WT enlargements a stream of eight Purkinje cell is shown.

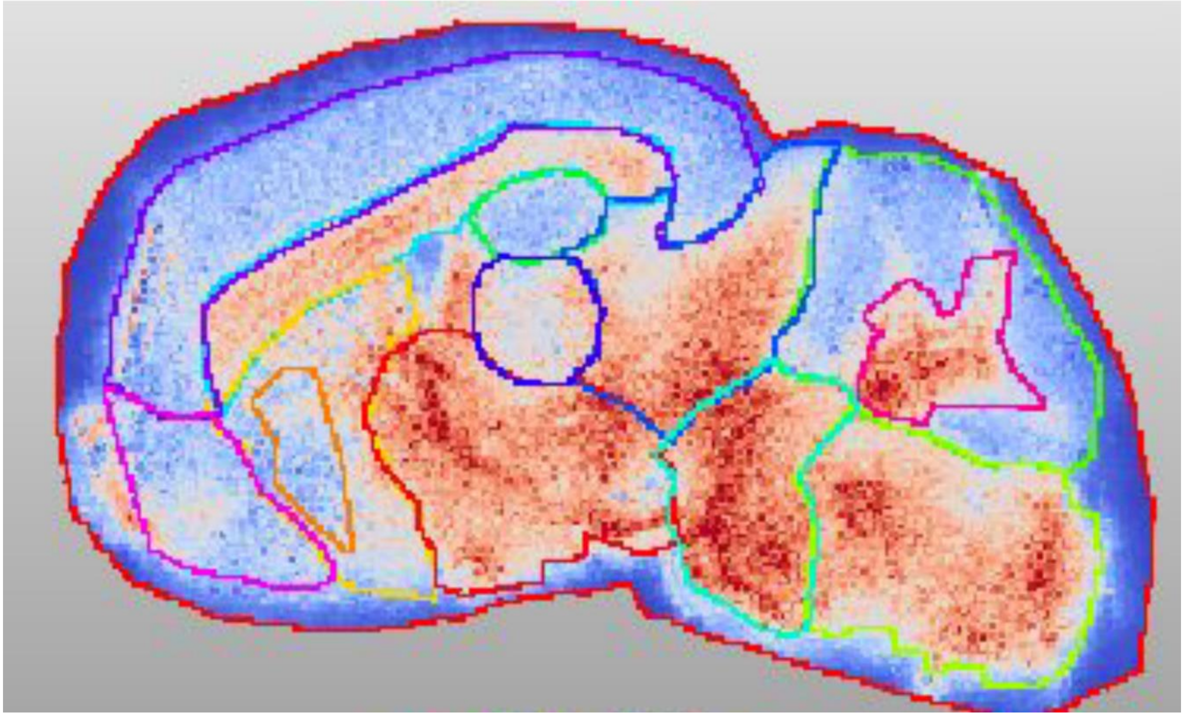

Supplemental Figure S7: Example of definition of ROI outlines in SCiLS Lab software (SCiLS, Bremen, Germany). Data were acquired on a vacuum-MALDI-TOF MS.

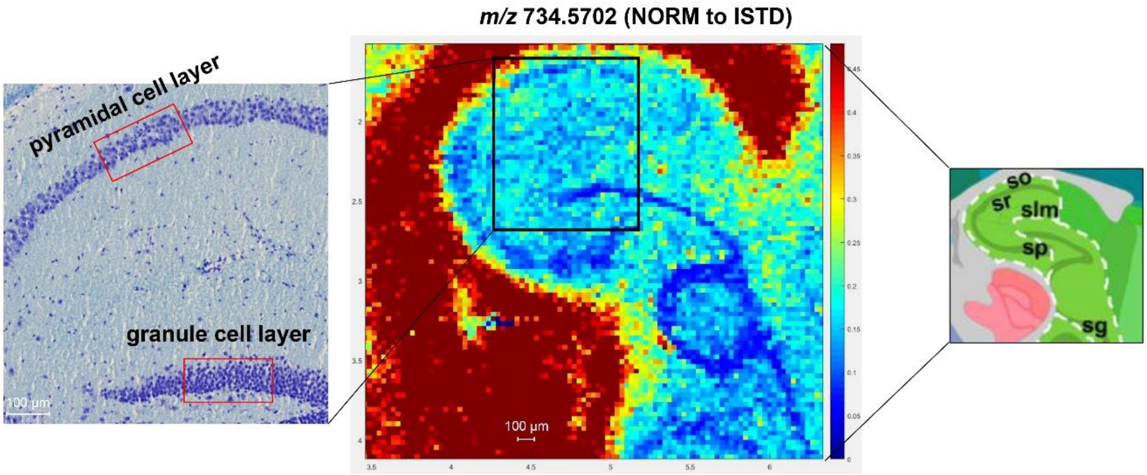

Supplemental Figure S8: High spatial resolution of fine hippocampal structures via AP-MALDI-Orbitrap. Centre panel, AP-MALDI-Orbitrap MSI of PC 32:0 in sagittal sections of WT adult mouse brain after on-tissue EADSA. Left panel, LFB/CV stain of a section adjacent to MSI with scale-bar 100 µm. Red rectangles in left panel are 200 x 100 µm. Right panel, reference anatomy with abbreviations as in Supplemental Figure S3.

The granule cell layer (sg) presents from 4 to 8 neurons (y-dimension) with cell bodies of 10–18 µm in diameter, and the pyramidal layer (sp) 3 to 4 neurons (y-dimension) with substantially larger cell bodies (~20 µm in diameter)[5]. In the selected region outlined in the red box, the granule cell layer measures ~90 µm in thickness, where we can count nuclei of about 7 small neurons (y-dimension). In our MSI measurement, the thickness of this layer is about 3 pixels = 90 µm.

Similarly, across the selected region of the pyramidal layer (sp) in the red box, we can count 3-4 larger neurons (y-dimension) and the layer is about 75 µm thick. This also agrees with our MSI measurement, where the thickness of this layer is about 2-3 pixels = 75 µm. These measurements are in good agreement with other reports, see for example Figure 3A and B in Ayuob et al[6].

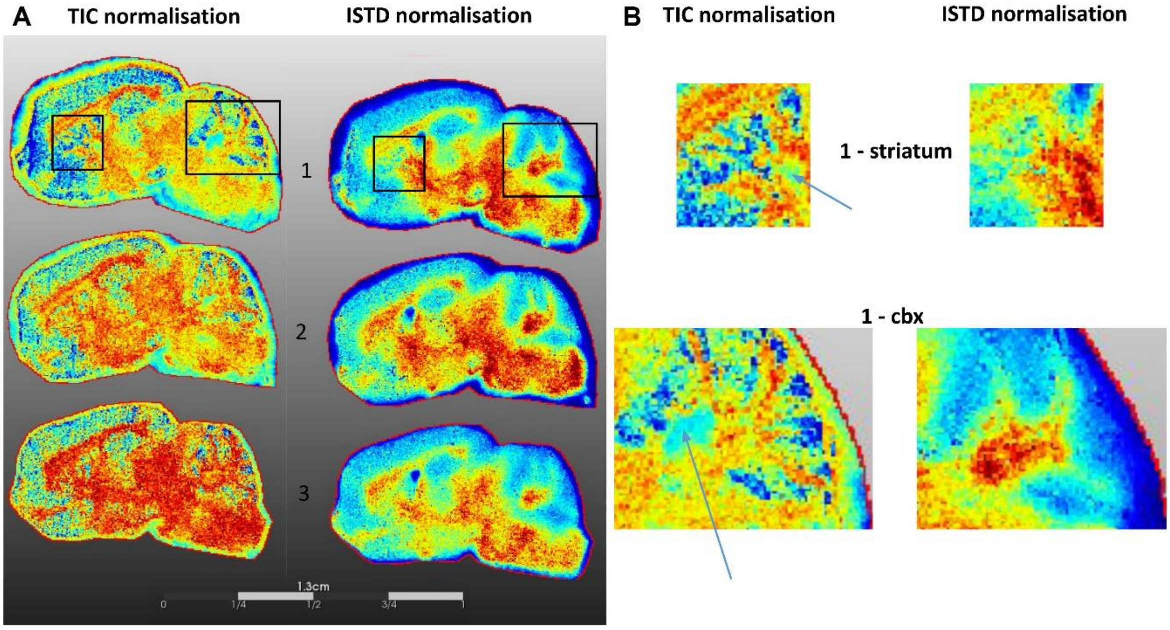

Supplemental Figure S9: Comparison of MSI normalisation strategies i.e. total ion current (TIC) vs isotope-labelled standard (ISTD). (A) MSI of cholesterol in adjacent sagittal sections of a WT mouse brain. The three replicates are indicated by consecutive numbering; on the left images are normalised to TIC while on the right images are normalised to ISTD. (B) 3X-enlargements of the striatal region of the caudate-putamen (above) and of the cerebellum (below). Data were acquired on a vacuum-MALDI-TOF MS.

Both normalization strategies reveal similar patterns in cholesterol abundance for macro-structures. However, enlargements (3X) in panel B shows that small subregions of the brain enriched in cholesterol are more clearly visualized with in the ISTD-normalized image than with TIC normalization.

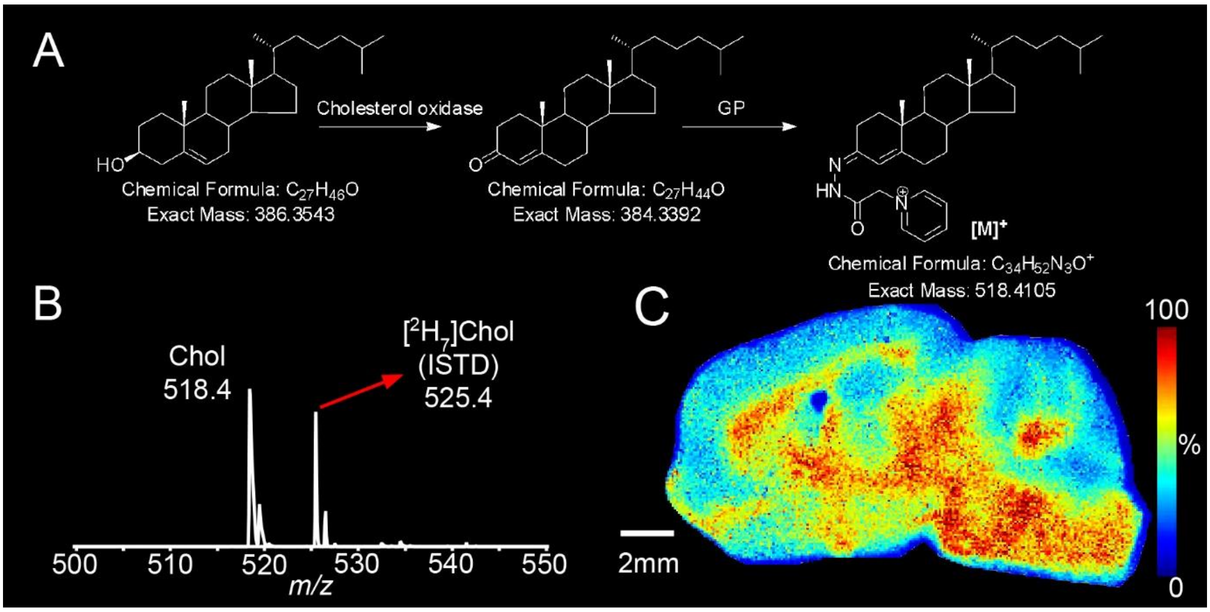

Supplemental Figures S10: A larger version of Figure 1.

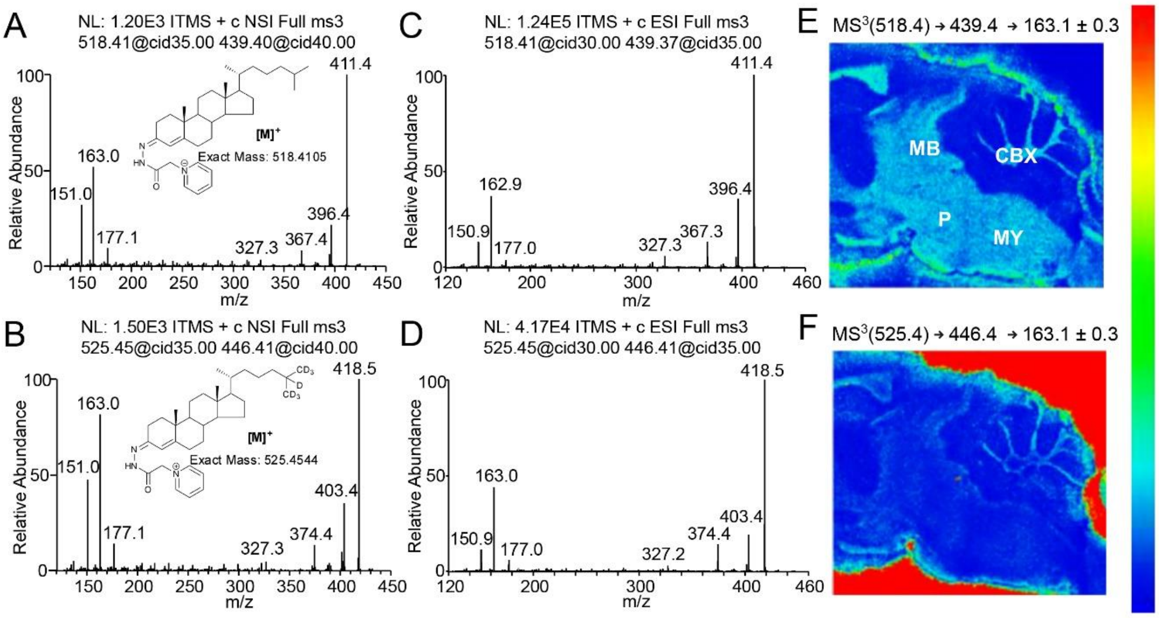

Supplemental Figures S11: A larger version of Figure 2.

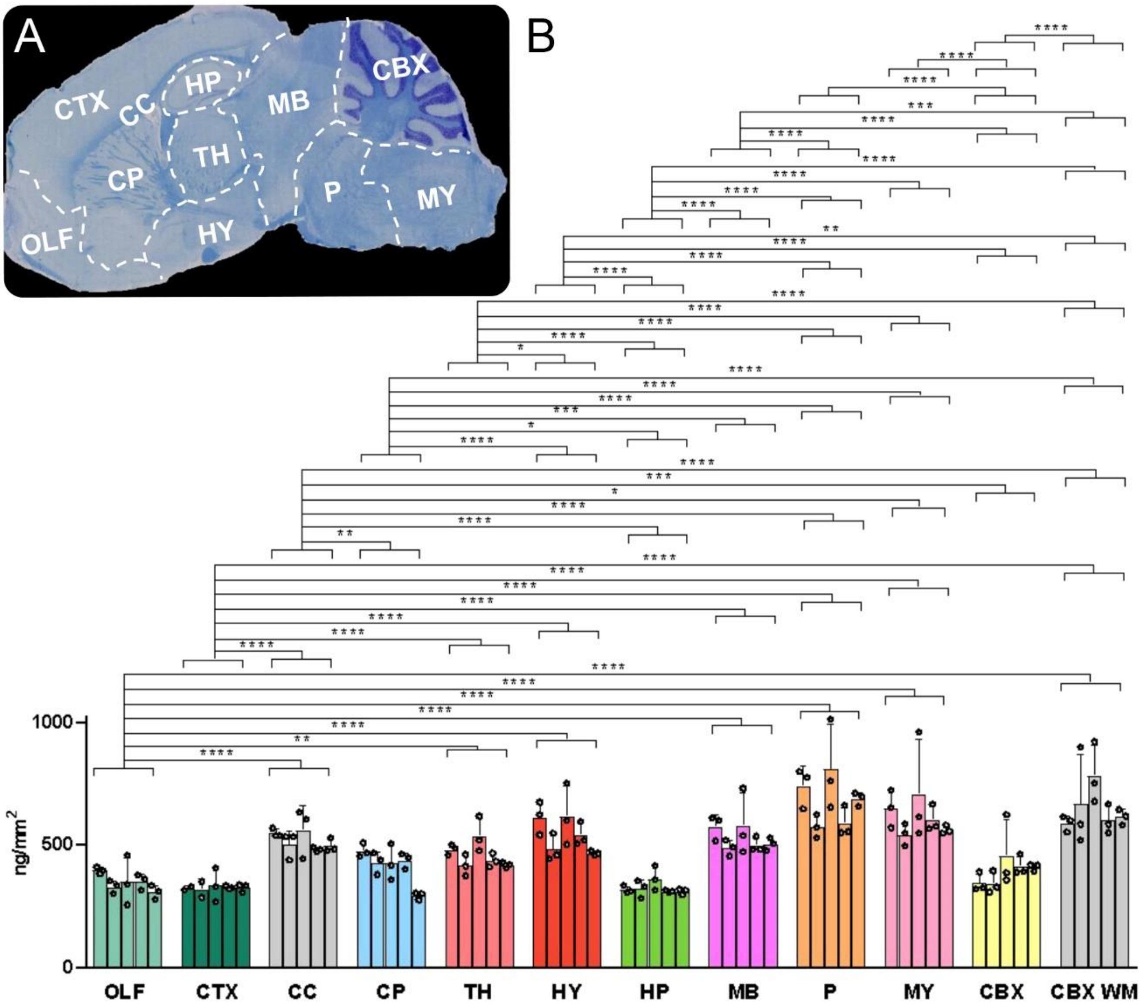

Supplemental Figures S12: A larger version of Figure 3.

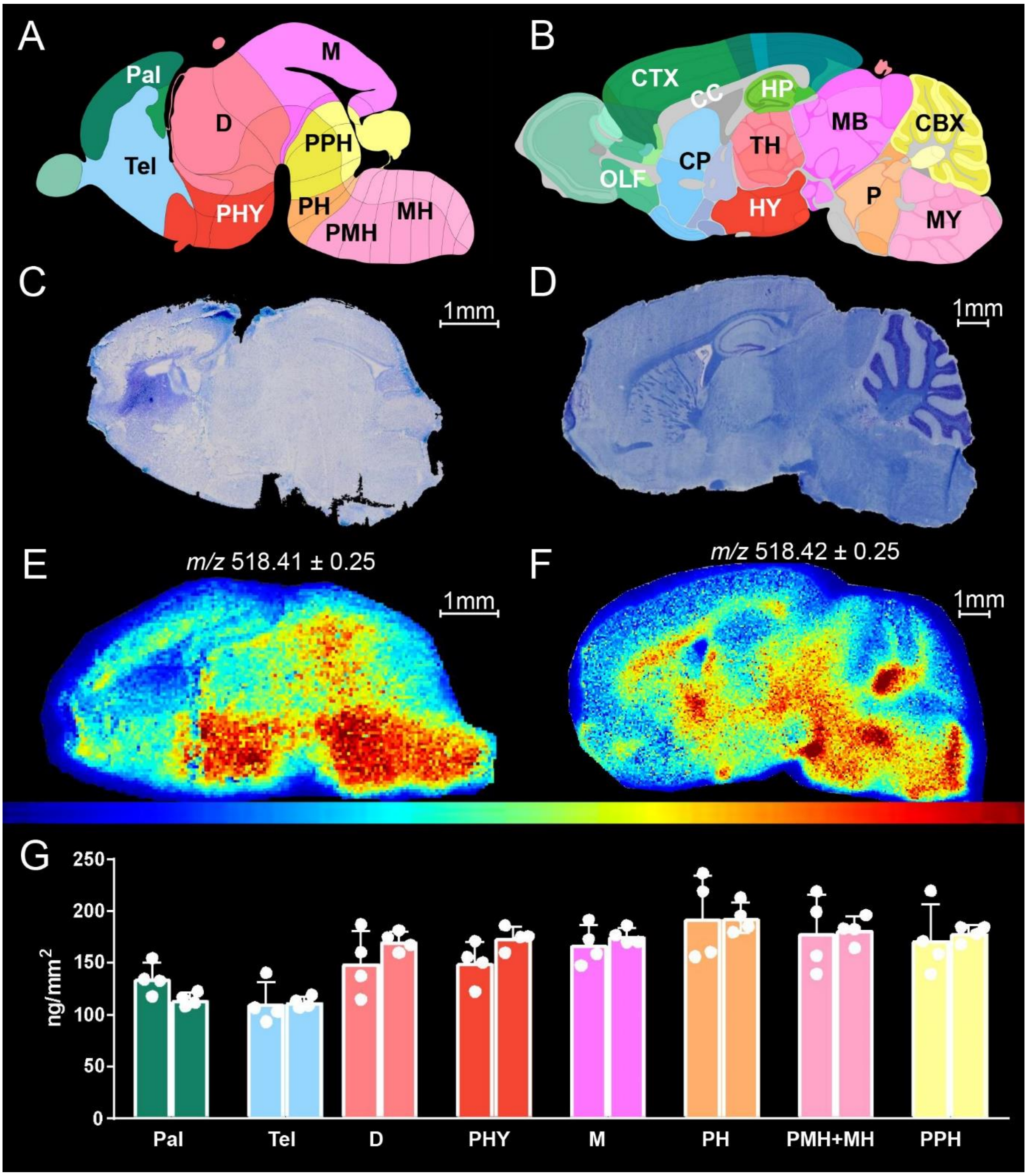

Supplemental Figures S13: A larger version of Figure 5.

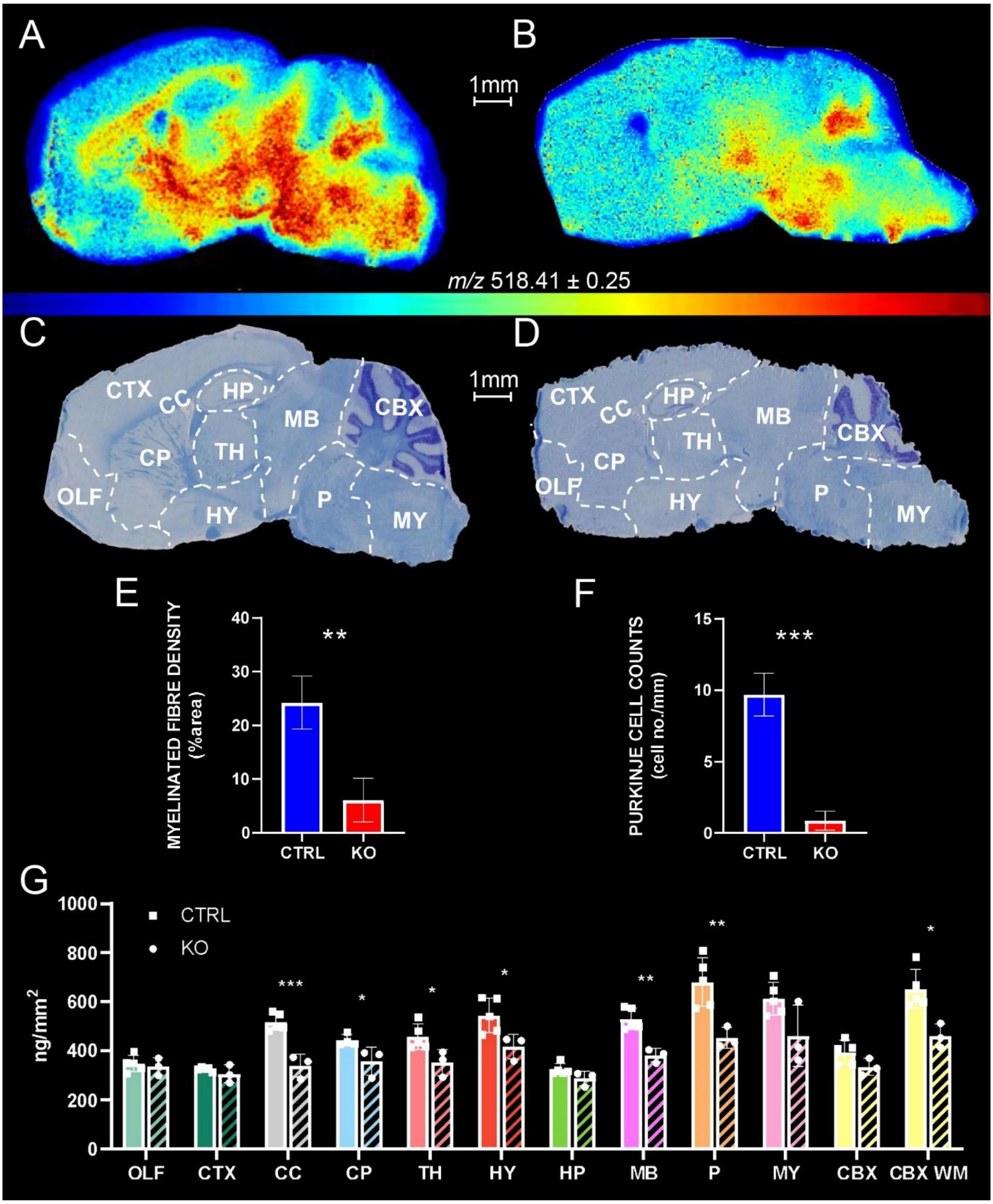

Supplemental Figures S14: A larger version of Figure 6.

Supplemental Table S1 Instruments used to produce the images shown and purpose of the experiments.

Figure #	MS Instrument	Purpose of the experiment
1	Vacuum-MALDI-TOF (Bruker ultrafleXtreme)	Demonstrate on-tissue EADSA
2	AP-MALDI-LIT-Orbitrap (ThermoFisher Scientific Orbitrap Elite)	MS^3 identification of cholesterol
3	Vacuum-MALDI-TOF (Bruker ultrafleXtreme)	Quantitative MSI in WT mouse
4	AP-MALDI-LIT-Orbitrap (ThermoFisher Scientific Orbitrap Elite)	Detection of phospholipids + high lateral resolution
5	Vacuum-MALDI-TOF (Bruker ultrafleXtreme)	Quantitative MSI in developing mouse
6	Vacuum-MALDI-TOF (Bruker ultrafleXtreme)	Quantitative MSI in *Npc1*$^{-/-}$ mouse
S2	Vacuum-MALDI-TOF (Bruker ultrafleXtreme)	Obtaining calibration curves
S3	AP-MALDI-LIT-Orbitrap (ThermoFisher Scientific Orbitrap Elite)	High lateral resolution of fine brain structures
S4	Vacuum-MALDI-TOF (Bruker ultrafleXtreme)	Corroborating quantitative MSI
S5A	Vacuum MALDI-Q-IM-TOF (Waters Synapt G2-Si)	Assaying robustness of method
S5C	DESI-Q-IM-TOF (Waters Synapt G2-Si)	Assaying robustness of method
S5E	DESI-Q-IM-TOF (Waters Synapt G2-Si)	Assaying robustness of method
S7	Vacuum-MALDI-TOF (Bruker ultrafleXtreme)	ROI outlines
S8	AP-MALDI-LIT-Orbitrap (ThermoFisher Scientific Orbitrap Elite)	High lateral resolution of fine brain structures
S9	Vacuum-MALDI-TOF (Bruker ultrafleXtreme)	Comparing normalizations

Supplemental Table S2 Cholesterol areal density values in defined brain regions of WT and *Npc1*$^{-/-}$ adult mice. In the present MALDI study n = 5 WT and n = 3 *Npc1*$^{-/-}$ mice (10-weeks of age) were employed (three sections per mouse); in the LESA study n = 3 WT mice (12 weeks of age). Mean ± standard deviation is reported together with percentage agreement between MALDI- and LESA-MSI measurements of cholesterol levels in WT mouse brain, as are percentage differences in brain cholesterol levels as measured by MALDI MSI in WT and in *Npc1*$^{-/-}$ mice.

Brain area	MALDI MSI WT (ng/mm^2)	LESA MSI WT (ng/mm^2)	MALDI Vs LESA WT (% Agreement)	MALDI MSI *Npc1*$^{-/-}$ (ng/mm^2)	MALDI MSI WT Vs *Npc1*$^{-/-}$ (% Difference)
olfactory traits	348.5 ± 52.4			337.5 ± 35.2	3.2
cortex	327.8 ± 32.5	305.1 ± 53.8	93.1	307.4 ± 36.9	6.2
corpus callosum	519.2 ± 55.9			340.8 ± 44.6	34.4
caudate-putamen	414.0 ± 74.1	366.8 ± 4.7	88.6	357.5 ± 50.7	13.6
thalamus	458.9 ± 59.2	446.8 ± 51.7	97.4	352.6 ± 51.9	23.2
hypothalamus	545.7 ± 89.1			417.7 ± 50.2	23.5
hippocampus	326.3 ± 31.6	252.9 ± 27.3	77.5	288.9 ± 36.5	11.5
mid brain	530.6 ± 70.6	356.7 ± 74.8	67.2	383.1 ± 32.0	27.8
pons	681.6 ± 123.9	575.5 ± 122.0	84.4	454.2 ± 49.5	33.4
medulla	613.8 ± 111.5	421.3 ± 61.6	68.6	461.6 ± 111.3	24.8
cerebellum	395.0 ± 76.7			335.8 ± 40.0	15.0
cerebellar white matter	652.0 ± 119.8	509.2 ± 83.0	78.1	462.1 ± 58.7	29.1

Supplemental Table S3 Cholesterol areal density values in defined brain areas of two WT newborn mice as quantified by MALDI-MSI. Four replicate sections per each mouse were employed. Averages ± standard deviation across all replicates are reported.

Brain area	Cholesterol (ng/mm^2)
pons	125.1 ± 15.3
diencephalon	160.5 ± 24.0
mid brain	171.9 ± 14.1
prepontine hindbrain	175.6 ± 23.2
pontine hindbrain	193.4 ± 28.4
pontomedullary and medullary hindbrain	180.3 ± 25.6
peduncular hypothalamus	162.2 ± 19.8
telencephalic vesicle	111.7 ± 13.9

Supplemental References:

1. Tobias, F.; Olson, M. T.; Cologna, S. M. Mass spectrometry imaging of lipids: untargeted consensus spectra reveal spatial distributions in Niemann-Pick disease type C1. *J. Lipid Res.* **2018,** *59*, 2446-2455.
2. Correa-Cerro, L. S.; Wassif, C. A.; Kratz, L.; Miller, G. F.; Munasinghe, J. P.; Grinberg, A.; Fliesler, S. J.; Porter, F. D., Development and characterization of a hypomorphic Smith–Lemli–Opitz syndrome mouse model and efficacy of simvastatin therapy. *Human Molecular Genetics* **2006,** *15* (6), 839-851.
3. Kluver, H.; Barrera, E., A method for the combined staining of cells and fibers in the nervous system. *J Neuropathol Exp Neurol* **1953,** *12* (4), 400-403.
4. Lein, E. S.; Hawrylycz, M. J.; Ao, N.; Ayres, M.; Bensinger, A.; Bernard, A.; Boe, A. F.; Boguski, M. S.; Brockway, K. S.; Byrnes, E. J.; Chen, L.; Chen, L.; Chen, T.-M.; Chi Chin, M.; Chong, J.; Crook, B. E.; Czaplinska, A.; Dang, C. N.; Datta, S.; Dee, N. R.; Desaki, A. L.; Desta, T.; Diep, E.; Dolbeare, T. A.; Donelan, M. J.; Dong, H.-W.; Dougherty, J. G.; Duncan, B. J.; Ebbert, A. J.; Eichele, G.; Estin, L. K.; Faber, C.; Facer, B. A.; Fields, R.; Fischer, S. R.; Fliss, T. P.; Frensley, C.; Gates, S. N.; Glattfelder, K. J.; Halverson, K. R.; Hart, M. R.; Hohmann, J. G.; Howell, M. P.; Jeung, D. P.; Johnson, R. A.; Karr, P. T.; Kawal, R.; Kidney, J. M.; Knapik, R. H.; Kuan, C. L.; Lake, J. H.; Laramee, A. R.; Larsen, K. D.; Lau, C.; Lemon, T. A.; Liang, A. J.; Liu, Y.; Luong, L. T.; Michaels, J.; Morgan, J. J.; Morgan, R. J.; Mortrud, M. T.; Mosqueda, N. F.; Ng, L. L.; Ng, R.; Orta, G. J.; Overly, C. C.; Pak, T. H.; Parry, S. E.; Pathak, S. D.; Pearson, O. C.; Puchalski, R. B.; Riley, Z. L.; Rockett, H. R.; Rowland, S. A.; Royall, J. J.; Ruiz, M. J.; Sarno, N. R.; Schaffnit, K.; Shapovalova, N. V.; Sivisay, T.; Slaughterbeck, C. R.; Smith, S. C.; Smith, K. A.; Smith, B. I.; Sodt, A. J.; Stewart, N. N.; Stumpf, K.-R.; Sunkin, S. M.; Sutram, M.; Tam, A.; Teemer, C. D.; Thaller, C.; Thompson, C. L.; Varnam, L. R.; Visel, A.; Whitlock, R. M.; Wohnoutka, P. E.; Wolkey, C. K.; Wong, V. Y.; Wood, M.; Yaylaoglu, M. B.; Young, R. C.; Youngstrom, B. L.; Feng Yuan, X.; Zhang, B.; Zwingman, T. A.; Jones, A. R., Genome-wide atlas of gene expression in the adult mouse brain. *Nature* **2007,** *445* (7124), 168-176.
5. Amaral, D. G.; Scharfman, H. E.; Lavenex, P., The dentate gyrus: fundamental neuroanatomical organization (dentate gyrus for dummies). *Prog Brain Res* **2007,** *163* (3-22), 788-790.
6. Ayuob, N. N.; L. Firgany, A. E.; El-Mansy, A. A.; Ali, S., Can Ocimum basilicum relieve chronic unpredictable mild stress-induced depression in mice? *Exp. Mol. Pathol.* **2017,** *103* (2), 153-161.
